# Advanced applications in chronic disease monitoring using IoT mobile sensing device data, machine learning algorithms and frame theory: a systematic review

**DOI:** 10.3389/fpubh.2025.1510456

**Published:** 2025-02-21

**Authors:** Yu Liu, Boyuan Wang

**Affiliations:** ^1^Hefei University of Technology, Hefei, China; ^2^Department of Biomedical Sciences, City University of Hong Kong, Hong Kong, China; ^3^Beijing Xiaotangshan Hospital, Beijing, China

**Keywords:** the internet of things, artificial intelligence, machine learning, deep learning, chronic disease

## Abstract

The escalating demand for chronic disease management has presented substantial challenges to traditional methods. However, the emergence of Internet of Things (IoT) and artificial intelligence (AI) technologies offers a potential resolution by facilitating more precise chronic disease management through data-driven strategies. This review concentrates on the utilization of IoT mobile sensing devices in managing major chronic diseases such as cardiovascular diseases, cancer, chronic respiratory diseases, and diabetes. It scrutinizes their efficacy in disease diagnosis and management when integrated with machine learning algorithms, such as ANN, SVM, RF, and deep learning models. Through an exhaustive literature review, this study dissects how these technologies aid in risk assessment, personalized treatment planning, and disease management. This research addresses a gap in the existing literature concerning the application of IoT and AI technologies in the management of specific chronic diseases. It particularly demonstrates methodological novelty by introducing advanced models based on deep learning, tight frame-based methodologies and real-time monitoring systems. This review employs a rigorous examination method, which includes systematically searching relevant databases, filtering literature that meets specific inclusion and exclusion criteria, and adopting quality assessment tools to ensure the rigor of selected studies. This study identifies potential biases and weaknesses related to data collection, algorithm selection, and user interaction. The research demonstrates that platforms integrating IoT and machine learning algorithms for chronic disease monitoring and management are not only technically viable but also yield substantial economic and social advantages in real-world applications. Future studies could investigate the use of quantum computing for processing vast medical datasets and novel techniques that merge biosensors with nanotechnology for drug delivery and disease surveillance. Furthermore, this paper examines recent progress in medical image reconstruction, emphasizing tight frame-based methodologies. We discuss the principles, benefits, and constraints of these methods, assessing their efficacy across diverse application contexts.

## 1 Introduction

### 1.1 The challenges and opportunities in public health management of chronic diseases

Cardiovascular diseases, cancer, chronic respiratory diseases, and diabetes constitute the most prevalent chronic diseases globally (1). According to data from the World Health Organization, cardiovascular diseases are the leading cause of non-communicable disease deaths, accounting for an estimated 17.9 million fatalities annually. This is followed by cancer with 9 million deaths, respiratory diseases with 3.9 million deaths, and diabetes with 1.6 million deaths. These four categories of diseases collectively account for 80% of all non-communicable disease fatalities worldwide (2). In China, over 80% of annual mortality is attributed to chronic non-communicable diseases such as heart disease, stroke, hypertension, and diabetes (3). The Global Burden of Disease study published in The Lancet further emphasizes that chronic diseases have emerged as the predominant disease burden globally, necessitating immediate preventative and control measures (4).

Challenges in monitoring and managing chronic diseases encompass limitations in the scope of surveillance, a lack of universality in prevention strategies, weak individual self-management skills, an absence of supportive policy environments, disconnects between projects and routine operations, a deficiency in scientifically robust project evaluation mechanisms, inadequate coordination across institutional tiers, the imperative to enhance residents' health literacy, human resource constraints, and a rising trend of young individuals contracting chronic diseases (5). These challenges compromise the precision, continuity, and comprehensiveness of chronic disease surveillance data, thereby impeding the effective deployment of prevention and control strategies and the optimal health management of patients with chronic diseases. Furthermore, the substantial disease burden imposed by chronic conditions, coupled with pervasive unhealthy lifestyles, further complicates the task of chronic disease prevention and control (6). To address these issues, it is essential to implement comprehensive measures that incorporate big data and information technology to refine chronic disease management, fortify health education initiatives, optimize the policy environment, and bolster coordination among medical institutions at various levels.

Traditional methods of chronic disease monitoring predominantly rely on manual processes, which are constrained by a limited scope of population surveillance, data collection limitations, accuracy concerns, and a lack of universality in prevention and management strategies (7). These methods are further compromised by weak self-management capabilities and compliance issues that affect the effectiveness of monitoring and management. Additionally, challenges such as the inconvenience of accessing medical services, the phenomenon of information silos, the absence of real-time monitoring and feedback, uneven distribution of medical resources, outdated technology and methods, and the lack of effective prediction and early warning mechanisms further impede traditional monitoring methods (8). However, with the advancement of information technology, modern tools such as electronic health records, mobile health applications, telemedicine, and big data analysis are gradually mitigating these limitations and enhancing the efficiency and effectiveness of chronic disease monitoring (9). For instance, the collection of individual health data through mobile health management devices and its upload to cloud platforms not only enhances data accessibility and timeliness but also aids in improving risk prediction and management of chronic diseases (10). Furthermore, the application of machine learning technology has become crucial for analyzing large medical and health datasets, widely used in early disease prediction, diagnosis, and prognosis assessment. This has significantly improved the accuracy and practicality of chronic disease risk prediction models (11).

IoT is a network concept that encompasses various networks, including the Internet, traditional telecommunication networks, and sensor networks. It facilitates the formation of an interconnected network by enabling all ordinary physical objects to be independently addressed, thereby achieving intelligent identification, positioning, tracking, monitoring, and management (12). IoT mobile sensing devices, which include a variety of wearable devices, portable medical devices, and embedded sensors, can be utilized to collect physiological and behavioral data from patients with chronic diseases (13). Machine learning, an AI technology that enables computer systems to continuously improve by learning from data and patterns, thus enabling them to make predictions or decisions without explicit programming (14), is another key component. The integration of IoT technology and machine learning algorithms offers new possibilities for real-time monitoring, risk assessment, and personalized treatment of chronic diseases.

In the realm of real-time monitoring, Mishra et al. have developed an automated intelligent lung cancer detection model based on the Health IoT. This model employs a Greedy Best-First Search algorithm and a Random Forest classifier to continuously monitor and collect patient data, thereby assisting clinical personnel in the early identification of disease risks associated with lung cancer and enhancing the accuracy of lung cancer diagnosis. Chronic disease management requires the collection and analysis of a vast amount of health data (15). Abubeker et al. have developed a wearable blood glucose monitoring (iGM) system supported by IoT. This wearable iGM device can continuously monitor the blood glucose levels of patients, transforming diabetes care and improving the quality of life for patients (16). Dhanasekaran et al. have developed a novel Multi-Objective Water Wave Optimization (MOWWO) algorithm. This algorithm utilizes Support Vector Machines (SVM) to handle the massive amounts of chronic disease data generated by IoT and employs wearable devices in telemedicine systems, such as wearable ultrasound patches, for cluster-based healthcare monitoring, thereby improving the diagnostic efficiency of chronic diseases like cardiovascular diseases (17).

In the realm of risk assessment, Liao et al. employ a method that involves processing, cleaning, and filtering data gathered from IoT sensors such as wearable electrocardiogram monitors, smart health watches, and blood pressure monitors in the cloud. They utilize artificial neural networks (ANN), in combination with genetic algorithms and error backpropagation mechanisms, to scrutinize electronic clinical data pertaining to the patient's medical history. This approach enables them to accurately diagnose the risk of heart disease (18). Similarly, Yashudas et al. proposed a cardiovascular disease prediction recommendation system based on an IoT network. This system employs four types of biosensors—electrocardiogram sensors, stress sensors, pulse sensors, and glucose sensors—to remotely collect physiological data from patients. The collected data is then used to assess the risk of heart disease, thereby providing patients with early diagnosis, treatment, and dietary recommendations (19).

In the realm of personalized treatment, Nanehkaran et al. proposed a medical recommendation system that utilizes IoT devices to identify and treat chronic diseases. This system employs the K-Nearest Neighbors (KNN) classification method to determine the type of disease and uses collaborative filtering to identify the most effective treatment for patients. The results suggest that this approach offers superior accuracy in diagnosing and predicting chronic diseases (20). Casillo et al. developed an IoT-based framework that incorporates machine learning techniques to collect precise data from patients with respiratory and cardiovascular diseases using wearable devices such as smartwatches (e.g., heart rate or blood oxygen levels). This framework provides a comprehensive view of treatment progress to monitor hydrotherapy care, thereby offering personalized customer management for tailored treatment (21). Baseer et al. introduced a novel coronary artery disease prediction model that integrates medical IoT with AI. This model captures real-time data from interconnected medical devices, wearables, and sensors, including continuous heart rate monitoring, electrocardiograms, and blood pressure readings. The model employs a combination of TabNet and catBoost to process and interpret the complex data obtained through the medical IoT, thereby achieving personalized cardiovascular disease risk assessment (22).

**Figure 1:**
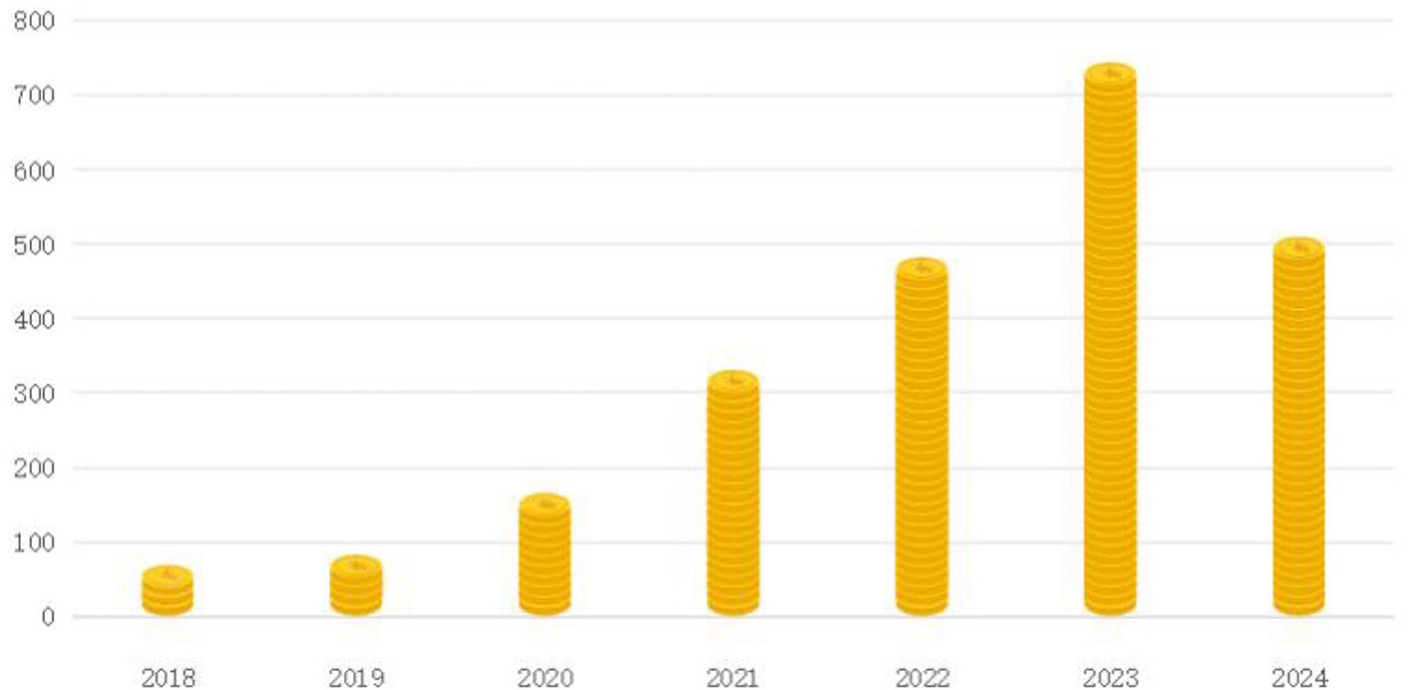
Distribution of the number of relevant research papers.

Despite the abundance of reviews on IoT-enabled chronic disease monitoring, as shown in Figure 1, our literature review reveals a significant gap in studies that exclusively focus on the evolution of machine learning (ML)-based techniques for big data analysis in the IoT healthcare sector. Our manuscript aims to fill this gap by providing a comprehensive analysis of ML techniques and their applications in IoT-enabled smart healthcare systems. The rapid advancement in IoT and ML technologies has led to the development of innovative solutions for chronic disease management. Our manuscript provides an up-to-date review of these advancements, highlighting the latest trends and their potential impact on healthcare management. Our review not only discusses theoretical aspects but also provides practical applications and case studies that demonstrate the real-world implementation and effectiveness of IoT and ML in chronic disease monitoring. This practical perspective is crucial for healthcare practitioners and policymakers.

Our manuscript offers an exhaustive analysis of various ML techniques, including deep learning, reinforcement learning, and traditional ML algorithms, which are less frequently covered in a single survey. This comprehensive coverage provides a holistic view of the current state of ML in IoT healthcare. Unlike other surveys that may focus solely on the advantages of ML techniques, our manuscript critically evaluates the strengths and weaknesses of existing ML techniques in the context of IoT healthcare. This balanced analysis helps readers understand the limitations and potential improvements needed. We highlight various research challenges and suggest future directions in the field, which can serve as a roadmap for researchers and developers working on IoT-enabled chronic disease monitoring systems. Our manuscript takes an interdisciplinary approach by integrating insights from healthcare, data science, and engineering, providing a multifaceted perspective that is often missing in specialized surveys. We have conducted an extensive literature review, including the most recent publications up to 2024, ensuring that our manuscript reflects the latest developments and research findings in the field.

Our paper provides an in-depth analysis of various ML techniques applied to big data in the context of IoT-enabled smart healthcare systems. This includes a review of both traditional and advanced ML algorithms, offering a broad perspective on their capabilities and applications. Through our systematic literature review, we have identified significant research gaps in the field, particularly regarding the integration of ML-based big data analytics in IoT healthcare. Our paper highlights these gaps, which can guide future research directions. We critically evaluate the strengths and weaknesses of existing ML techniques, providing a balanced view that can inform the selection of appropriate techniques for specific healthcare applications. Our review includes practical applications and case studies that demonstrate the real-world implementation of IoT and ML in chronic disease monitoring, offering insights into their effectiveness and potential for improvement. We propose future research directions, addressing the challenges and opportunities in the field, which can serve as a roadmap for researchers and practitioners in the development of IoT-enabled chronic disease monitoring systems.

We commend the studies that employed robust methodologies, such as large-scale multi-center cohort studies and rigorous machine learning algorithms, which have contributed to the reliability and validity of their findings. We acknowledge the innovative use of IoT devices and advanced machine learning models that have pushed the boundaries of chronic disease monitoring and management, offering new insights into patient care. We critically assess the generalizability of the study results, noting where sample sizes and demographic representations may limit the applicability of findings to broader populations. We address concerns regarding data accuracy, noting instances where IoT devices may introduce biases, and where machine learning algorithms may not be representative of specific populations, affecting the precision of chronic disease risk predictions. We discuss the ethical implications of data collection and storage, especially in the context of IoT and AI technologies, and the measures taken by studies to ensure patient privacy and data security. We highlight areas where the current literature is lacking and suggest directions for future research to build upon the existing body of knowledge.

This study addresses a gap in the existing literature concerning the application of IoT and AI technologies in the management of specific chronic diseases. It particularly demonstrates methodological novelty by introducing advanced predictive models based on deep learning and real-time monitoring systems. Our systematic review employs a rigorous examination method, which includes systematically searching relevant databases, filtering literature that meets specific inclusion and exclusion criteria, and adopting quality assessment tools to ensure the rigor of selected studies. This research identifies potential biases and weaknesses related to data collection, algorithm selection, and user interaction. For example, certain IoT devices may have biases in data accuracy, while some machine learning algorithms may lack representativeness for specific populations. The unique perspective addressed in this paper is evaluating the effectiveness of specific IoT devices in managing cardiovascular diseases and comparing the accuracy of different machine learning algorithms in predicting chronic disease risks, especially among the older adult population. The research demonstrates that platforms integrating IoT and machine learning algorithms for chronic disease monitoring and management are not only technically viable but also yield substantial economic and social advantages in real-world applications. This highlights their potential as future paradigms for chronic disease management.

### 1.2 The potential of IoT and AI in chronic disease management

The integration of IoT and AI technologies presents substantial potential in the realm of chronic disease management. These technologies not only augment the efficiency of management processes and enhance medical diagnostic capabilities, but also foster patients' self-management skills and overall quality of life. The emerging approach of data-driven chronic disease management utilizes IoT devices to gather data, and employs machine learning algorithms for comprehensive analysis. The objective is to achieve a more precise management of chronic conditions.

Giannakopoulou et al. gather data pertaining to Parkinson's patients through the use of smart devices, wearable or non-wearable sensors, and other Internet of Things (IoT) technologies. They employ machine learning and deep learning methodologies to offer support for both Parkinson's patients and healthcare providers at every stage of the disease, thereby optimizing therapeutic outcomes and minimizing medical costs (23). Abdel-Fattah et al. proposed a hybrid machine learning technique that is based on a big data platform (Apache Spark). This technique utilizes feature selection methods and classification algorithms such as decision trees, logistic regression, naive Bayes, random forests, and gradient-boosted trees to collect and analyze data related to chronic kidney disease. This approach provides timely feedback and medical interventions for patients, thereby enhancing their quality of life (24). Lee et al. employs stepwise logistic regression, decision trees, random forests, and SVM, among other machine learning techniques, to rapidly process the vast amounts of data collected by IoT devices such as electrocardiogram monitors. This provides summaries and descriptions of the health status of older adult patients with chronic diseases like diabetes and cerebral palsy, thereby enhancing the accuracy of chronic disease diagnosis (25). Symum and Zayas-Castro uses five algorithms, including decision trees, linear support vector machines (LSVM), KNN, random forests, and multi-layer artificial neural networks, in conjunction with semi-supervised anomaly detection and two feature selection methods to construct models. These models predict the trends and potential complications of diseases such as congestive heart failure, acute myocardial infarction, and chronic obstructive pulmonary disease, thereby enhancing diagnostic capabilities (26). Tian et al. employs the use of SVM to conduct normative analysis on data derived from Parkinson's, multiple sclerosis, stroke, and other diseases. This is achieved through the utilization of smart devices such as pulse oximeters. The methodology provides data-driven recommendations and decision support, thereby enhancing the quality and efficiency of medical services (27).

The amalgamation of IoT and AI in chronic disease management presents novel strategies for refining the management process and elevating patient quality of life. This integration facilitates superior patient education, intelligent monitoring, and digital empowerment at the primary level of chronic disease management. Jimenez et al. detailed the endeavors undertaken within the European TeNDER project, which focuses on chronic diseases among the older adult population. TeNDER is a system tailored for older adult individuals with chronic conditions such as Alzheimer's disease, Parkinson's disease, and cardiovascular diseases. It employs a sensory ecosystem to enable patients to monitor their health status at all times, thereby enhancing their quality of life (28). Morales-Botello et al. leveraged emerging technologies such as big data, cloud computing, and IoT, in combination with medical guidelines and knowledge bases, to offer patients with chronic diseases like cardiovascular diseases, hypertension, and chronic obstructive pulmonary disease educational information on disease knowledge, medication information, lifestyle, scientific monitoring, and complications. This methodology has significantly improved the quality of life for patients (29). Yu et al. introduced an AI-centric system for chronic disease management, integrating AI, knowledge graphs, big data, and IoT on a unified platform. This system autonomously incorporates follow-up data into patients' health records, generates follow-up reports, and alerts physicians for necessary interventions, thereby enhancing compliance among pediatric chronic disease patients (30). Singh et al. merged fog computing with AI and smart health to offer a robust platform for the early detection of thyroid infections and to maintain comprehensive health records throughout the disease trajectory. By assimilating in-hospital data, public health information, and patient monitoring records from external sources, the system automatically aggregates this data into structured charts, thereby elevating medical management efficiency (31). Omboni et al. employed Tholomeus, a remote medical solution rooted in medical IoT, to boost patient medication adherence via intelligent reminders and medication logs. By synergizing big data for chronic disease management, electronic medical records, and IoT technologies, the system ensures that patients do not miss pivotal moments for chronic disease management, thus enhancing overall management efficacy (32).

In the realm of chronic disease management, our primary focus is on the long-term monitoring and treatment of persistent conditions such as hypertension and diabetes. The advent of technology has led to a significant role for medical image analysis in this field. This analysis aids physicians in achieving more precise diagnoses, evaluating treatment outcomes, and formulating personalized treatment plans for patients. For example, in diagnosing stroke, medical image processing techniques can scrutinize magnetic resonance imaging (MRI), and computed tomography (CT) images, underscoring the importance of early diagnosis for patient treatment and recovery. As the need for medical image analysis in chronic disease patients escalates, so too does the potential for advancements in medical image reconstruction technology. Particularly, deep learning-based methods are becoming instrumental in enhancing imaging quality, reducing radiation doses, and improving patient care. Deep learning image reconstruction (DLR) technology represents one of the most advanced developments in MRI image reconstruction. It surpasses traditional MRI image reconstruction techniques by delivering superior quality images, thereby providing doctors with more accurate diagnostic information. Moreover, recent research progress in AI for medical CT image reconstruction suggests that the use of feature pyramid networks, GPU-accelerated image reconstruction, and other technologies can significantly enhance the efficiency and quality of image reconstruction.

Chronic disease image reconstruction constitutes a significant area of research in contemporary medical imaging, aiming to restore comprehensive medical images from partial or compromised data. As medical imaging technologies, such as MRI and CT, continue to advance, the requirements for image quality and resolution escalate correspondingly. Consequently, the effective reconstruction of high-quality medical images has emerged as a central focus of research attention. In recent years, the application of tight frame theory has significantly expanded in the realm of image processing and analysis, particularly within the domain of image reconstruction. Tight frames serve as an efficacious mathematical instrument for articulating the sparsity and structural attributes of images. When integrated with other sophisticated methodologies such as compressed sensing, total variation, and deep learning, tight frames introduce novel opportunities and challenges in the field of medical image reconstruction. Numerous studies have explored medical image reconstruction methods based on tight frames. These methodologies address various challenges, including managing uneven intensity, reconstructing CT images from limited angles, and leveraging data-driven approaches to enhance the quality of MRI reconstructions. Moreover, some research endeavors seek to amalgamate tight frames with alternative techniques to further optimize reconstruction outcomes. Despite the advancements in tight frame-based medical image reconstruction, numerous challenges persist. These include determining the optimal choice of tight frames, addressing intricate noise and artifacts, and ensuring the clinical relevance of the reconstructed images.

The novelties of this review are predominantly reflected in the ensuing aspects:

(1) Interdisciplinary integration: the review skillfully amalgamates insights derived from health care, data science, and engineering disciplines, thereby providing a comprehensive perspective that is frequently absent in expert surveys.
(2) Comprehensive literature review: the review offers an exhaustive review of the literature, incorporating the most recent publications from 2024, to ensure that the latest advancements and research findings in the field are accurately reflected.
(3) Emphasis on deep learning-based models: this review places significant emphasis on the exploration and understanding of sophisticated models grounded in deep learning.
(4) Real-time monitoring systems: the review introduces IoT and ML algorithm-based monitoring and management platforms for chronic diseases. These platforms are not only technically viable but also offer significant economic and social advantages in real-world applications.
(5) Data-driven chronic disease management: the review investigates the potential utility of IoT devices for data collection, coupled with machine learning algorithms for comprehensive data analysis, thereby enabling more precise management of chronic diseases.
(6) Multimodal datasets and methodologies: review that encompass a range of ML techniques, such as DL, RL, and conventional machine learning algorithms, offers an exhaustive perspective on the prevailing state of ML within the sphere of IoT healthcare.
(7) Application of tight frame: the review discusses the application of tight frame theory to augment the restoration and reconstruction of medical images. It introduces a data-driven tight frame magnetic resonance imaging reconstruction method (DDTF-MRI), alongside a space-Radon domain CT image reconstruction model employing data-driven tight frames (SRD-DDTF).
(8) Utilization of quantum computing in healthcare: this review proposes possible applications of quantum computing in the management of extensive medical data. It also explores emerging technologies in drug delivery and disease monitoring, achieved through the integration with biosensors.

## 2 The application of key IoT and AI technologies in chronic disease management

### 2.1 The application of IoT in chronic disease management

In the realm of lifestyle monitoring, the use of IoT mobile sensing devices for tracking the diet, exercise, and sleep patterns of chronic disease patients is on the rise. These devices include smart wearables, non-contact biometric radar devices, and remote monitoring kits. In the field of remote health monitoring, IoT mobile sensing devices are instrumental in the continuous tracking of vital signs in chronic disease patients. This includes real-time monitoring and data transmission, non-contact monitoring, and predictive maintenance of medical devices, among others.

Portable medical devices, typically battery-powered and handheld, are compact medical instruments that are easy to transport and operate. Zaman and Morshed introduced a rechargeable, battery-operated portable scanner (i.e., demodulator) for collecting data from body-worn wireless resistive analog passive sensors. This device can obtain biological signals such as body temperature, electrocardiogram (ECG), oxygen saturation, electromyogram (EMG), and respiratory rate from patients (33). Anh Tran Tam Pham et al. developed a portable medical open platform for fluorescence measurements at varying excitation and emission wavelengths. This platform is used to detect changes in the level of albumin in urine samples, thereby aiding in the detection and monitoring of chronic kidney disease (34). Mateen et al. employed a portable magnetic resonance scanner to provide basic vital sign monitoring data, which is instrumental in the detection of demyelinating diseases of the central nervous system (35).

Wearable medical devices, such as smartwatches and health tracking bands, are designed to be worn directly on the body. These devices can monitor and record physiological parameters in real-time, including heart rate, blood pressure, and blood sugar levels. They also utilize software support to perform data analysis and provide health recommendations. Vybornova et al. developed an IoT-based, non-invasive, cuffless blood pressure monitor that measures optical photoplethysmographic pulse wave signals on the wrist. This device calculates systolic and diastolic pressure values using pulse wave analysis technology (36). Grandner et al. evaluated the performance of a new IoT-based device, the Happy Ring, which assesses sleep continuity and structure. The personalized algorithm of this device demonstrated higher sensitivity and detection accuracy compared to general methods and other devices (37). Wu et al. proposed an IoT-based real-time health monitoring system that uses deep learning. This system employs wearable medical devices to measure vital signs of patients with chronic diseases such as brain tumors, heart disease, and cancer. It applies various deep learning algorithms to extract valuable information, assisting doctors in accurately analyzing patient conditions (38).

Ingestible medical devices, also known as ingestible capsule endoscopes and drug delivery systems, are small electronic devices that patients can swallow. These devices are typically used for monitoring and drug release within the digestive tract. van der Schaar et al. employed remotely controlled ingestible drug delivery devices to target medication delivery to specific areas of the intestine, thereby treating various diseases of the small intestine (39). Weitschies et al. utilized swallowable sensors to gather physiological data from the patient's gastrointestinal tract. This data is crucial for the successful development of drug products, which can be used for the diagnosis of inflammatory bowel diseases (40).

Implantable medical devices, such as cardiac pacemakers and implantable cardioverter-defibrillators (ICDs), necessitate surgical insertion into the human body. These devices can monitor physiological conditions over extended periods or deliver treatments like neurostimulators or drug pumps. Yacoub and McLeod pioneered the development of these implantable devices, notably the ICDs, which are instrumental in detecting impending unstable heart failure or pulmonary hypertension, thereby significantly reducing the readmission rate among patients with chronic heart failure (41). Similarly, Israel et al. successfully implanted pacemakers equipped with atrial fibrillation detection and electrogram storage features in patients exhibiting physiological pacing indications and a history of atrial fibrillation. They regularly monitored these patients for symptoms related to atrial fibrillation and optimized their antiarrhythmic drug therapy (42).

Intelligent clinical devices primarily cater to medical staff, including mobile nursing management, infusion monitoring management, smart wards, and bedside intelligent interaction. These devices assist nursing personnel in obtaining real-time patient information and enhancing nursing efficiency. Shamsabadi et al. employed various IoT sensors to monitor and track vital signs of type 2 diabetes patients, such as heart rate, blood pressure, oxygen saturation, and body temperature. This approach facilitated physicians in managing patients virtually via the internet, thereby improving treatment methods and overall patient health (43). Sandhu and Singh developed an automatic medication dispenser that can be easily controlled through a mobile application. This product ensures the management of medication intake timing and dosage through a mobile application, thereby reducing the burden on healthcare personnel (44). Naseem et al. proposed an IoT-supported electrocardiogram monitoring system. This system can calculate statistical features of raw ECG signals and employ the Pan Tompkins QRS detection algorithm to assess the signals. The system is used to extract heart rate variability characteristics for the diagnosis of arrhythmia diseases (45). Wei et al. designed a new type of IoT-based non-contact device called ultra-wideband bio-radar. This device can detect respiratory signals through bio-radar and diagnose sleep apnea diseases. The analysis of its monitoring results is automated, eliminating the need for manual scoring and scorers (46).

Remote health devices, such as wireless monitoring platforms, are capable of performing a variety of functions including remote dynamic blood pressure monitoring and remote wireless health check management. These devices also facilitate the establishment of personal electronic health records and enable the conduct of health management and interventions. Wu et al. proposed a comprehensive and scalable remote precision health service aimed at promoting health and preventing chronic diseases. This service integrates wearable devices, open environmental data, indoor air quality sensing devices, location-based smartphone applications, and AI-assisted remote nursing platforms to achieve continuous real-time monitoring of lifestyle and environmental factors (47). Chang et al. utilized IoT-based sensors for the remote monitoring of chronic diseases, including the detection of patient fall risks, epileptic seizures, or pressure sores. These sensors were connected to internet medical services to provide a range of remote medical services, such as remote monitoring, remote consultation, and robot-assisted surgery (48). Chatrati et al. proposed a smart home health monitoring system that enables the remote analysis of patients' blood pressure and blood sugar indices at home. This system predicts the status of hypertensive and diabetic patients by combining conditional decision-making and machine learning, and notifies healthcare providers when any abnormalities are detected (49).

### 2.2 The application of algorithms from classical statistics, machine learning, and deep learning in disease diagnosis and management

We have designed a hybrid model that combines the strengths of different machine learning algorithms to predict chronic disease outcomes. This model incorporates a deep learning component for feature extraction and a classical machine learning component for classification, offering a more accurate and nuanced analysis. Utilizing the data from the reviewed papers, we have created a risk assessment tool that can identify patterns and trends in chronic disease management. This tool employs a novel feature selection process to prioritize the most influential factors in disease progression. We have simulated a real-time monitoring system using IoT data, which allows for the testing of our machine learning models in a dynamic environment. This simulation provides insights into how these models could perform in actual clinical settings. We have developed a predictive analytics model that forecasts the impact of various interventions on chronic disease outcomes. This model uses historical data to predict future trends, offering a proactive approach to disease management. To ensure the reliability of our findings, we have incorporated a set of quality assessment tools and bias mitigation techniques. These tools help to identify and adjust for potential biases in the data, ensuring that our conclusions are robust and valid.

The volume of data in the medical field is escalating, necessitating an urgent need for data processing and analysis. Presently, most medical research hinges on statistical data, with the interpretation of vast amounts of health-related information largely reliant on the chosen statistical methods and how these data are employed to test hypotheses and estimate associations (50). The role of statistics in research and clinical practice has become increasingly integral (51). Medical statistics extends beyond mere data collection and analysis; it elucidates complex relationships between disease occurrence, prognosis, treatment effects, and their associated factors through systematic methodologies (52). Table 1 presents a comprehensive overview of various chronic disease datasets utilized in the application of statistical and machine learning methodologies for disease diagnosis and management. These datasets encompass a range of diseases, including diabetes, cardiovascular diseases, and chronic kidney disease, highlighting the diversity and volume of data available for research and analysis. This information is crucial for developing effective predictive models and improving patient outcomes through data-driven approaches (53). To enhance the understanding and application of medical statistical data, researchers and medical professionals have dedicated themselves to developing and implementing various methods and technologies. For instance, Mishra et al. employed numerical and visual methods to assess the normality of biomedical research data (54). Benvenuto et al. employed fast unconstrained Bayesian approximation analysis to determine that the nucleocapsid and spike glycoproteins exhibit sites under positive pressure. Additionally, their homology model revealed distinct molecular and structural differences among viruses (55). Liang and Kelemen introduced univariate time-varying Bayesian state-space models and multivariate Bayesian state-space models. They integrated various prior models with hyper-prior models using the Markov Chain Monte Carlo (MCMC) algorithm to estimate model parameters and hidden variables. Their findings indicated that these proposed models effectively predicted genomic dynamics behavior (56). Liang and Kelemen developed a Bayesian finite Markov mixture model with a Dirichlet prior to identify differentially expressed time-related genes and dynamic clusters. This model adeptly captured the dynamic changes and patterns of irregular complex time data (57). Dufault et al. suggested a multi-indicator flexible Bayesian framework to facilitate efficient mid-trial decision-making in multi-arm multi-stage phase II clinical trials (58). Chen et al. proposed a class of semi-parametric transformation models with log-normal frailty. They employed the expectation-maximization algorithm in conjunction with a screening method to approximate infinite-dimensional parameters for estimating all parameters, analyzing data sets from rodent carcinogenicity experiments (59). Xu et al. applied non-parametric maximum likelihood estimation for inference and evaluated these methods in terms of asymptotic properties, simulation studies, and a randomized clinical trial of nasopharyngeal carcinoma (60). Thao et al. investigated two models to assess the influence of quantified risk factors on disease outcomes: the Cox proportional hazards model, which incorporates death as a competing risk, and the disease-death model that perceives the disease as a potential intermediate state. Their findings revealed that the disease-death model, evaluated at the penultimate visit, exhibited superior performance across all simulated environments (61). At present, traditional statistical methods, machine learning, and deep learning have emerged as indispensable tools in medical research and clinical practice.

**Table 1:** Chronic disease datasets used in statistical and machine learning analysis.

**Dataset name**	**Region**	**Index**	**Disease**	**Amount of data**	**References**
Pima Indian Diabetes Dataset	India	Plasma Glucose Concentration	Diabetes	968	(250)
UCHTT1DM	Chile	Glucose, Heart Rate, IGAR, Step Count, etc.	Diabetes	20	(251)
Sylhet Diabetes Dataset	Bangladesh	Weight, Eyesight, Food Intake, etc.	Diabetes	520	(252)
Tehran Diabetes Dataset	Iran	Body Mass Index and Working Heart Rate	Diabetes	3,376	(253)
National Institute Diabetes Dataset	America	Glucose, Blood Pressure, Skin Thickness, etc.	Diabetes	768	(254)
OCTA and Fundus Images Multimodal Dataset	India	Retinal Images	Non-Proliferative Diabetic Retinopathy	222	(255)
Radiology Dataset	/	Heart Medical Images	Cardiovascular Diseases	1 lakh	(256)
Cleveland Clinic Heart Disease	America	Resting Blood Pressure, Serum Cholesterol, Fasting Blood Sugar, etc.	Cardiovascular Diseases	303	(257)
The Framingham Heart Study Dataset	America	Body Mass Index, Level of glucose, etc.	Coronary Heart Disease	4,240	(258)
NIH Chest X-ray Dataset	America	Smoking, Alcohol Intake, etc.	Heart-Related Diseases	122,120	(259)
PASCAL Classifying Heart Sound Challenge Dataset	/	Heart Sound, etc.	Heart Disease	400	(259)
Jordan University Hospital Heart Dataset	Jordan	Blood Pressure, Pulse, etc.	Heart Disease	486	(260)
Tawan Hospital CKD Dataset	United Arab Emirates	Cholesterol Levels, Triglyceride Levels, etc.	CKD	544	(261)
Salford Kidney Study Dataset	Britain	Single Nucleotide Polymorphisms	CKD	1,919	(262)
Parkinson's Disease Smartwatch Dataset	Germany	Wrist Movements, etc.	Parkinson's Disease	469	(263)

Classical statistical methods are extensively utilized in medical research. These techniques emphasize data collection and analysis, with inferences drawn through model establishment and hypothesis testing. For instance, Luo et al. employed a blend of Cox proportional hazards regression and log-rank tests to pinpoint significant prognostic factors for predicting the overall survival of drugs, thereby enhancing predictive performance by 4% (62). Similarly, Barnett-Itzhaki et al. leveraged classical statistics, specifically logistic regression, to forecast *In vitro* fertilization (IVF) outcomes based on various parameters such as the number of retrieved oocytes, mature oocytes, good quality embryos, positive β-hCG, clinical pregnancy, and live birth. The accuracy of this prediction consistently ranged between 0.34 and 0.74 (63). Pires and Rodrigues introduced two distinct methodologies for estimating pertinent parameters of linear models: one employing maximum likelihood under the assumption of normal errors and another incorporating results from robust linear regression. The latter method is designed to counteract distant observations or error distributions characterized by heavy tails, yielding the most precise results for the analyzed dataset (64). Broderick et al. employed partial least squares (PLS) to develop two feature spaces, utilizing multiplicative scatter correction and quantile normalization to eliminate trends and adjust ranges in microarray data. Their findings indicated that the distinction between individuals and non-fatigued subjects was underpinned by two co-regulation patterns, accounting for 10% of the total microarray variation (65). Brentnall et al. utilized the Tyrer-Cuzick model for breast cancer risk assessment and prediction, estimating the risk ratio of the highest decile of 10-year risk relative to the middle 80% of the study population (66). While classical statistical methods offer interpretability and stability, they are limited by assumptions about data distribution and constraints on data volume. Furthermore, these methods often necessitate model construction based on prior knowledge and assumptions, which can be challenging when dealing with complex medical data.

With the burgeoning advancements in computer science, machine learning has emerged as a pivotal tool in the realm of medicine. Khafaga et al. employed data mining techniques, including Adaboost and random forest, to enhance the precision of clinical decision-making in the hemodynamic assessment of abdominal aortic aneurysms (67). Ilyas et al. determined that the J48 decision tree algorithm outperformed the random forest in detecting chronic kidney disease (CKD) stages, suggesting the potential utility of an automated system based on this model for CKD severity detection (68). Althnian et al. identified Adaboost and Naive Bayes as the most resilient models when confronted with limited medical data, highlighting that model efficacy is contingent upon the dataset's representation rather than its volume (69). Mishra et al. observed that the Correlation Feature Selection (CFS) method was superior in terms of accuracy and execution time for chronic disease prediction, while Best-First Search (BFS) distinguished itself among all wrapper (boost) methods. The proposed hybrid approach, which integrated enhanced K-means clustering, CFS filtering, and BFS wrapper methods, achieved optimal classification performance across various chronic disease datasets (70). Sidey-Gibbons and Sidey-Gibbons devised three predictive models for cancer diagnosis—regularized generalized linear model regression, support vector machine with radial basis function kernel, and single-layer artificial neural network—utilizing descriptions from nuclei extracted from breast lumps. Their findings indicated that the algorithm could classify nuclei with high accuracy (0.94–0.96), sensitivity (0.97–0.99), and specificity (0.85–0.94) (71). Battineni et al. investigated the use of SVM in predicting dementia, with performance validated through statistical analysis. The findings revealed an accuracy and precision rate of 68.75% and 64.18%, respectively (72). Xing and Bei introduced an enhanced KNN algorithm that incorporates cluster denoising and density trimming. This improved algorithm demonstrated a notable enhancement in classification efficiency when processing large medical health datasets, while preserving the original classification accuracy of the KNN algorithm (73). Palaniappan employed respiratory sounds from the R.A.L.E database to assess and contrast the efficacy of SVM and KNN classifiers in diagnosing respiratory pathologies. The outcomes revealed classification accuracies of 92.19% for the SVM classifier and 98.26% for the KNN classifier (74). Alanazi suggested a method that utilizes machine learning algorithms, including Convolutional Neural Network (CNN) and KNN, to detect and forecast the onset of chronic diseases in individuals. This approach yielded an accuracy rate of 95%, surpassing other algorithms such as Naive Bayes, decision trees, and logistic regression (75). Pourhomayoun and Shakibi developed a predictive model utilizing machine learning algorithms to assess the health risks of COVID-19 patients and predict mortality. Their comparisons indicated that a neural network model could achieve an impressive accuracy rate of 89.98% (76). Dahiwade et al. employed KNN and CNN algorithms for disease prediction, with results showing that the CNN algorithm had a higher accuracy rate of 84.5% compared to the KNN algorithm (77). Hatwell et al. utilized the Adaptive Weighted High Importance Path Segment (Ada-WHIPS) algorithm to assist medical practitioners in making critical decisions regarding patient conditions. Experimental results on relevant datasets demonstrated that Ada-WHIPS had superior generalization capabilities (average coverage of 15%−68%) compared to existing technologies, and also outperformed in specificity (average precision of 80%−99%) (78). Tang et al. employed AdaBoost and random forests algorithms, which exhibited excellent classification performance (accuracy over 95%) in enhancing tissue pathology decisions using infrared spectroscopy (79). Machine learning algorithms have proven effective in automatically learning and extracting useful features from large volumes of medical data, and using these features for prediction and decision-making. This approach has yielded significant results in disease diagnosis, drug discovery, gene analysis, and medical image processing. Compared to traditional statistical methods, machine learning methods offer greater flexibility and adaptability, capable of handling more complex data patterns and relationships. However, the limitations of machine learning methods include their black-box nature and dependence on a large amount of labeled data.

Deep learning, a significant branch of machine learning, is increasingly being applied in the medical field. This technique, based on artificial neural networks, simulates human brain processes to recognize patterns and data features, thereby accomplishing complex tasks such as image and voice recognition, and natural language processing. Common deep learning models include CNN, Deep Belief Networks, and Stacked Autoencoder models. Saheed and Arowolo employed deep recurrent neural networks and supervised multi-labeling (SML) models to construct an efficient Intrusion Detection System (IDS) for the medical Internet of Things (IoMT) environment, achieving an impressive accuracy rate of 99.76% (80). Senan et al. discovered that hybrid models, which combine deep learning with machine learning, outperform standalone deep learning models. Specifically, the AlexNet (a CNN model) + SVM model demonstrated significant accuracy, sensitivity, and specificity in diagnosing Alzheimer's disease using magnetic resonance imaging (81). Zheng et al. proposed a deep learning-assisted Adaboost algorithm (DLA-EABA) for breast cancer detection, yielding an accuracy rate of 97.2%, a sensitivity of 98.3%, and a specificity of 96.5% (82). Reddy and Delen utilized RNN (Recurrent Neural Network)—LSTM (Long Short-Term Memory) to predict the readmission rate of lupus patients. The results indicated that the deep learning method RNN-LSTM outperformed traditional classification methods, with an AUC (Area Under the Receiver Operating Characteristic curve) of 0.70 (83). Dong et al. introduced a novel multi-task bidirectional recurrent neural network model, integrated with deep transfer learning, to enhance the performance of named entity recognition in electronic medical records under data constraints. This model surpassed baseline models, evidenced by a 2.55% increase in the micro-average F-score for discharge summaries and a 7.53% rise in overall accuracy (84). Banerjee et al. employed two deep learning models—CNN and Hierarchical Recursive Neural Network (DPA-HNN)—to synthesize information on pulmonary embolism from numerous free-text radiology reports of CT scans. The results indicated an optimal F1 score of 0.99 for the presence of pulmonary embolism (PE) in both adult and pediatric patient populations (85). Giunchiglia et al. proposed a recursive neural network model (RNN-SURV) for personalized survival analysis, achieving a superior concordance index (C-index) compared to state-of-the-art methods (86). Leevy et al. utilized RNN and Conditional Random Fields (CRF) methods for automatic de-identification of free-text, with their hybrid solution yielding the highest recall score of 94.16 compared to other methods tested (87). Gupta et al. proposed a generative adversarial network architecture to generate high-resolution medical images (88). Sun et al. employed generative adversarial networks (GANs) to share private medical image data, demonstrating good image fidelity, sample diversity, and dataset privacy (89). Sorin et al. used GANs to create artificial images for radiology applications, with the results indicating that the generated images enhanced the performance of the developed algorithms (90). Armanious et al. introduced a novel framework for generative adversarial networks (MedGAN) specifically tailored for medical image translation, demonstrating superior performance over existing translation methods through perceptual analysis and quantitative assessment (91). Tseng et al. employed GANs to extract the characteristics of patient populations necessary for training from a limited sample size, utilizing Deep Neural Networks (DNN) to reconstruct the Radiotherapy Artificial Environment (RAE) with both original and synthetic data generated by GAN. Their findings indicated that patients with retinitis pigmentosa 2 (RP2) had a normal tissue complication probability (NTCP) limit of 17.2% (92). Allesøe et al. developed a deep learning model for cross-diagnostic prediction of mental disorder diagnoses, revealing that this model exhibited strong predictive capabilities for diagnosis, with an AUC ranging between 0.71 and 0.82 (93). Xu et al. applied transfer learning of CNN and RNN to predict lung cancer treatment response, discovering a significant correlation between the CNN probability and the change in primary tumor volume (*P* = 0.0002) (94). Rajpurkar et al. leveraged deep learning to assist clinicians in diagnosing tuberculosis in human immunodeficiency virus (HIV) patients using chest X-rays, resulting in an average accuracy increase from 0.60 (95% CI 0.57, 0.63) to 0.65 (95% CI 0.60, 0.70) among clinical doctors (95). Kuo et al. utilized transfer learning technology to automate kidney function prediction and classification based on ultrasound-based kidney imaging, achieving an overall accuracy of 85.6% for chronic kidney disease state classification, surpassing that of experienced nephrologists (60.3%−80.1%) (96). Yoon et al. employed Deep Convolutional Neural Networks (DCNN) to identify occult scaphoid fractures, a task that is typically undetectable by human observers, with an accuracy rate of approximately 90% (97). Li et al. introduced a novel approach for the automatic interpretation of traditional transesophageal echocardiogram images and the intelligent guidance of probe motion, utilizing deep reinforcement learning technology. The results demonstrated that this method could effectively direct probe motion and exhibited strong generalization capabilities for invisible patient data (98). Kumar et al. applied Recurrent Neural Networks and reinforcement learning models to predict COVID-19, with the model's predictions aligning closely with the virus's state, achieving a correlation of 0.999 between the raw data and the predicted data (99). At the heart of deep learning lies the construction of multi-layer neural network models capable of automatically learning and processing complex medical data, extracting high-level features, and representations. This methodology has yielded groundbreaking results in medical image analysis, bioinformatics, and medical text processing. Deep learning methods are characterized by their strong generalization and learning capabilities, their ability to learn from large volumes of unlabeled data, and their exceptional performance in medical diagnosis and prediction. However, these methods also present challenges such as high computational resource demands, increased model complexity, and the need for data privacy protection. To further illustrate the diverse applications and comparative merits of various algorithms in disease prediction and health assessment, Table 2 provides a detailed comparative analysis of several recent studies.

**Table 2:** Comparative analysis of algorithms in disease prediction and health assessment.

**References**	**Proposed methodology**	**Merit**
Ahmed et al. (235)	This study explores the application of recursive feature cancellation and explainable AI-enhanced logistic regression models for predicting dementia.	- Enhancing the performance of linear regression by employing RFE technology. - Enhancing the interpretability of the model by employing SHAP values, which offer valuable insights into the features that exert the most influence on dementia prediction.
Osmani and Ziaee (236)	A decision tree learning algorithm was employed to evaluate the risk factors associated with vitamin D3 deficiency in patients suffering from chronic hepatitis B in the Birjand region.	- Utilizing data mining techniques to construct a decision tree model, which will facilitate the analysis of potential risk factors linked to vitamin D3 deficiency. - The efficacy of the model is assessed using Receiver Operating Characteristic (ROC) curves.
Kalita et al. (237)	This study aims to investigate the correlation between VDR polymorphisms and various types of HBV-related liver disease. Additionally, it seeks to develop a disease prediction model utilizing SVM.	- Investigating the correlation between VDR polymorphisms and HBV-associated liver disease. - Utilizing SVM models to forecast various disease stages.
Yousif et al. (238)	The early detection of chronic kidney disease is facilitated through the application of the Eurygasters optimization algorithm in conjunction with integrated deep learning methodologies.	- The detection and classification of CKD are achieved through a strategic approach that involves feature selection and hyperparameter tuning. - The EOAEDL-CKDD method was employed to experimentally evaluate the CKD dataset. The results indicated that the proposed method outperformed existing models in terms of detection rate.
Honarvar et al. (239)	This paper presents a deep learning-based tool for shear wave detection and segmentation, designed for clinical application in the assessment of chronic liver disease.	- Utilizing deep learning algorithms to detect and segment shear waves in liver tissue, thereby enhancing the precision of tissue characterization for patient diagnosis. - Software tools were integrated into the Velacur system to enhance the quality of liver assessments conducted by the operator.

### 2.3 Chronic disease prediction and early warning technologies

Upon utilizing machine learning techniques to analyze patients with chronic diseases, it becomes feasible to subsequently implement early warning systems and decision support mechanisms for the risk assessment of these conditions.

Sinha et al. applied ANN to biosensor data in order to predict chronic diseases such as chronic respiratory diseases and diabetes. They adjusted risk assessment models according to individual patient factors, including genetic background, lifestyle, and medical history, thereby achieving personalized predictions (100). Singh et al. utilized ANN-based models for the detection and early warning of chronic kidney disease, a model that surpassed SVM classifiers with an accuracy rate nearing 100% (101). Ma et al. introduced Heterogeneous Modified Artificial Neural Networks (HMANN) for the detection, segmentation, and diagnosis of chronic renal failure on Internet of IoMT platforms, demonstrating high precision in chronic disease risk assessment (102).

Lu et al. utilized the random forest model on extracted data features to predict the risk of type 2 diabetes, demonstrating superior performance compared to other models (103). Singh et al. integrated particle swarm optimization with the random forest for automated identification of various chronic diseases, thereby enhancing the precision of chronic disease risk assessment (104). Wang et al. combined the random forest model with logistic regression and other analyses to investigate urban-rural disparities and primary factors influencing depressive symptoms among the older adult in China, leading to improved accuracy and efficacy of treatment strategies (105).

Tu et al. developed a predictive model utilizing machine learning techniques, specifically SVM classifiers, to identify high-risk individuals for osteoporosis based on chronic disease data. They employed IoMT devices to gather patients' physiological and lifestyle information (106). Similarly, Wang and Wang employed models such as SVM to amalgamate information on chronic diseases like cardiovascular diseases and Parkinson's at the molecular level (i.e., genomics, epigenomics, proteomics, and metabolomics), along with clinical and laboratory data and environmental factors. They recommended personalized treatment plans based on risk assessment results and patient characteristics (107). Troosters et al. used machine learning algorithms, including SVM, to analyze the rehabilitation process of patients with chronic obstructive pulmonary disease (COPD). They adjusted the rehabilitation plan to align with the patient's recovery pace and needs, offering personalized rehabilitation guidance and support such as customized exercise plans and nutritional advice (108).

Chaudhuri et al. utilized Recursive Feature Elimination (RFE) to identify the most effective feature subset, and an ensemble algorithm known as boosted decision trees, to predict the risk of chronic diseases. The findings suggested that this model could significantly decrease both the time and error associated with treatment (109). Taser applied bagging and boosting techniques to experimental data for diabetes prediction using six distinct decision tree-based classifiers. The results indicated that the methods incorporating bagging and boosting achieved superior accuracy rates compared to individual decision tree classifiers (110).

Khalid et al. employed a variety of machine learning algorithms to evaluate the risk of chronic kidney disease, with gradient boosting demonstrating an accuracy rate of 99%, surpassing other algorithms (111). Theerthagiri and Vidya introduced a gradient boosting algorithm that utilizes Recursive Feature Elimination for precise heart disease risk assessment. They further scrutinized health records of patients exhibiting significant cardiovascular disease characteristics to pinpoint risk factors for the onset and progression of chronic diseases, as well as to forecast future health conditions of patients (112). Rufo et al. leveraged the principles of the Light Gradient Boosting Machine to construct an accurate diabetes diagnosis model. The experimental findings suggest that the compiled diabetes dataset is of considerable reference value for the early detection of diabetes in the Ethiopian region (113).

Hsieh et al. conducted a study on potential core acupoint combinations for the treatment of COPD by mining association rules from randomized controlled trials identified in prior meta-analyses, utilizing the Apriori algorithm-based association rule analysis (114). Zhang et al. applied the Apriori algorithm and multinomial logistic regression to investigate variations in multiple chronic disease patterns and associated factors among urban and rural older adult populations in China, offering a scientific foundation for developing health management strategies to mitigate urban-rural health disparities (115). Ma et al. employed association rule mining techniques to examine network association patterns between diseases presenting in the same individual, which can enhance prevention strategies, facilitate early identification of high-risk groups, and reduce mortality rates (116).

Sahu introduced a feature selection technique utilizing a genetic algorithm. This ensemble model classifier demonstrated superior accuracy on the CKD dataset compared to preceding and subsequent classification models, following the application of feature selection and dimensionality reduction techniques. The model is applicable for identifying CKD, evaluating treatment outcomes, monitoring rehabilitation progress, and dynamically adjusting risk assessment models and decision support systems based on monitoring results (117). Arabasadi et al. proposed an accurate hybrid diagnostic method for coronary artery disease, enhancing the initial weights of the neural network through a genetic algorithm. This improvement boosted the performance of the neural network by approximately 10% (118).

Utilizing the aforementioned machine learning techniques facilitates the early detection, timely warning, and personalized management of chronic disease risks. This approach significantly enhances patient treatment outcomes and overall quality of life.

### 2.4 Data security and privacy

Ensuring the privacy of health data for patients with chronic diseases is paramount, and this can be achieved through the application of data encryption and anonymization techniques.

Torfi et al. developed a differentially private framework for synthesizing chronic disease patient data, utilizing convolutional autoencoders and convolutional generative adversarial networks under the umbrella of generalized differential privacy. This model, within the same privacy budget, is capable of capturing temporal information and feature correlations inherent in the original patient data, thereby outperforming existing models in terms of patient privacy protection (119). Pitoglou et al. employed models such as logistic regression, decision trees, KNN, Gaussian naive Bayes, and SVM to the Mondrian algorithm with varying parameter values, generating anonymized clinical datasets of chronic disease patients (120). Ahmed and Kannan proposed that healthcare units implement a secure and privacy-preserving IoT integration to establish a reliable, available, and secure Remote Patient Monitoring (RPM) system for chronic disease patients. This system provides secure authentication based on Radio Frequency Identification (RFID), as well as end-to-end secure communication and privacy protection, achieving mutual authentication, user untraceability, prevention of replay attacks, forward and backward secrecy, and data integrity (121). Wenhua et al. is dedicated to designing a security model for managing data of chronic disease patients in healthcare systems. This involves using lightweight encryption algorithms in conjunction with patient IDs to generate access tokens, and strictly regulating access to patient departmental data to ensure the privacy and confidentiality of electronic health records in remote medical applications (122). Makina et al. conducted an extensive investigation into emerging research strategies aimed at addressing the security and privacy concerns of patients with chronic diseases. These strategies encompass cloud-based solutions, decentralized technologies such as blockchain and the InterPlanetary File System, encryption methods, and fine-grained access control policies (123). Akhbarifar et al. introduced a remote health monitoring model that employs lightweight block encryption techniques. This model utilizes data mining algorithms, including J48 decision trees, SVM, multilayer perceptrons, K-star, and random forests, to safeguard health and medical data within a cloud-based IoT environment, thereby ensuring the confidentiality of sensitive information for chronic disease patients (124). Oh et al. suggested a data sharing scheme for chronic disease medical information systems designed to preserve patient privacy. By combining Private Set Intersection (PSI) with K-anonymity, this scheme employs a single access key function to generate PSI. This enables data owners and users to determine if there is any common information in their respective private sets without revealing pertinent details (125). Trivedi and Patel proposed a framework that incorporates multiple intelligent chronic disease healthcare service providers and trusted third parties. This system is solely responsible for the dynamic authentication of chronic disease patients, eliminating the need to restart existing communication channels. By integrating dynamic tokens with secret sharing key updates, it ensures both privacy security and dynamic scalability (126). Babu et al. suggested a permissioned blockchain framework designed to securely exchange patient information pertaining to chronic diseases and to ensure the integrity of data sources. This system employs the Elliptic Curve Digital Signature Algorithm within the chronic disease healthcare blockchain network, thereby enabling nodes to interact anonymously and securely to share healthcare information within the data sharing network (127).

Regulatory compliance is an imperative for healthcare providers, necessitating a prioritization of data governance security. Policymakers face the challenge of aligning regulations with technological advancements, striking a balance between development and security, and ensuring data safety while fostering its development, utilization, and industrial progression. Data processing activities must adhere to laws and regulations, uphold social morality and ethics, operate in good faith, fulfill data security protection obligations, assume social responsibilities, and refrain from harming national security, public interests, or the legitimate rights and interests of individuals and organizations. Silva's recommendations encompass investing in robust data governance, staff education, promoting interoperability, ensuring ethical AI integration, and maintaining awareness and adaptability. The future of data sharing in the healthcare sector hinges on a commitment to ethical practices, regulatory compliance, and the seamless integration of advanced technology (128).

## 3 Smart healthcare and chronic disease management

### 3.1 Intelligent chronic disease management system

Mobile health, an innovative technology in the realm of chronic disease care, holds the potential to augment patients' self-management abilities, curtail healthcare expenditures, and elevate the quality of life. It offers a convenient and efficient method for chronic disease patients to manage their health via real-time monitoring, personalized services, and intelligent reminders. The role of intelligent diagnostic and treatment platforms is becoming increasingly significant in the diagnosis and monitoring of chronic diseases.

Wang et al. conducted a study on the influence of WeChat-based health management on the health and self-management efficacy of patients with severe chronic heart failure. The findings suggest that this approach can enhance the self-care ability and compliance of these patients, improve cardiac function and related indicators, reduce the incidence of cardiovascular adverse events, and prevent readmission (129). Weng et al. evaluated a virtual clinic platform in conjunction with specialized nursing care for symptomatic atrial fibrillation patients. This combination was found to increase patient satisfaction, quality of life, and the efficiency of emergency visits and hospitalizations (130). Zulfiqar et al. employed the intelligent MyPredi™ electronic platform to automatically detect the exacerbation of geriatric syndromes, including heart failure. The MyPredi™ platform is linked to a medical analytics system that receives and analyzes physiological data from IoMT sensors in real-time. Given the increased risks associated with geriatric diseases, the MyPredi™ remote monitoring platform has been shown to effectively mitigate these risks (131). Ali et al. assessed the effectiveness of Person-centered care for patients with chronic obstructive pulmonary disease and congestive heart failure by integrating a digital platform with structured telephone support (132).

Greene et al. used a performance platform to improve the clinical management of patients with diabetes and osteoporosis, resulting in an increase in osteoporosis screening rates among women (40% vs. 44%, *P* < 0.0001) (133). Shea et al. evaluated the longitudinal effect of Foodsmart, a digital nutrition platform that includes meal planning, meal ordering, and nutritional education features, on changes in glycated hemoglobin A1c levels in patients with diabetes. This reduction in A1c levels can help prevent other health complications and provides essential support for improving diet and blood sugar control in patients with diabetes (134).

Li et al. leveraged Hadoop, Spark, and data mining technologies to develop a comprehensive, real-time, intelligent mobile healthcare system. This system aids in the progressive detection and prediction of hypertension, offering a practical supplementary tool for self-directed user healthcare and enhancing the efficiency of patient disease diagnosis (135). Verweij et al. utilized the CMyLife platform to furnish chronic myeloid leukemia patients with tools and knowledge necessary to manage their care process. This improved medication adherence and molecular monitoring, thereby elevating the quality of life for these patients (136).

Inupakutika et al. proposed a concept for the development of an IoT-based mobile healthcare application aimed at supporting chronic patients. This concept integrates existing software platforms and services, thereby simplifying the development of various healthcare functionalities. It also empowers patients to self-monitor and manage their diseases and symptoms (137). Opipari-Arrigan et al. tested and utilized mobile health (mHealth) tools to promote the feasibility, acceptability, and short-term impact of a closed feedback loop and a vision of patient-clinician partnerships in chronic disease care models. The platform fosters collaboration between patients with inflammatory bowel disease and cystic fibrosis and clinicians through real-time, two-way data sharing, thereby improving strategies for pediatric chronic disease management and enhancing treatment efficiency (138). Doyle et al. designed and developed a digital health platform, ProACT, to facilitate the self-management of multiple conditions among older adults within the support of their care networks. This platform enables multi-morbid patients to self-manage multiple diseases on a single platform, assists users in understanding the relationship between their symptoms and conditions, provides personalized education targeted to individual health states, and supports data sharing with care networks (139). Guisado-Fernandez et al. aimed to create a multi-dimensional profile designed to monitor patients with dementia and support their informal caregivers at home. They also sought to conduct long-term follow-ups using the proposed wellbeing profile at different time intervals, thereby improving the quality of life for patients with dementia (140). Roca et al. proposed a chatbot architecture for chronic patient support, underpinned by three key pillars: scalability via microservices, standard data sharing through HL7 FHIR, and standard conversation modeling using AIML. This facilitates the interaction and collection of medical and personal information. A prototype specifically designed for psoriasis was suggested, offering significant enhancements in the development of chatbots as virtual assistants for chronic diseases (141). Reid et al. presented a novel platform that employs the Centers for Disease Control and Prevention's guidelines to remotely monitor patients with chronic respiratory diseases. This approach has been shown to reduce hospitalizations and emergency department visits, thereby improving patient quality of life and saving substantial healthcare costs (142). Ahmed et al. proposed an intelligent platform, GVViZ (Visualizing Genes with Pathogenic Variations), which is findable, accessible, interactive, and reusable. GVViZ can identify patterns and extract actionable information from millions of features, aiding in the early detection of diseases such as Alzheimer's and the development of new therapies for personalized patient care (143). Taylor et al. employed the Comparative Outcomes Real-World Research Platform (CONTOR) to conduct an exhaustive evaluation of patients suffering from irritable bowel syndrome with constipation and chronic idiopathic constipation. By amassing extensive longitudinal real-world data on the medical history, treatment experiences, and outcomes of IBS-C and CIC patients, CONTOR is able to gain a more profound understanding of this patient population (144).

### 3.2 Patient experience enhancement

The roles of AI and IoT technologies are escalating in the sphere of patient engagement and education, with a particular emphasis on disease management for chronic illness patients. Huang et al. employed IoT devices, such as smartwatches and health trackers, to develop a medication reminder system. This system comprised a pharmaceutical information cloud database, medical staff operating terminals, and patient terminals. The aim was to encourage patients to adhere to their medication schedules, undergo regular check-ups, and follow specific dietary and exercise plans (145). Kear et al. designed an integrated online and offline platform for chronic disease management. This platform facilitated the informatization of continuous medical services, including diagnosis, treatment, rehabilitation, post-diagnostic follow-up, and health education. The “Internet Plus” model was used to enhance patient compliance, reduce medical costs, improve the medical experience, and increase patient satisfaction (146). Treskes et al. utilized smart technology compatible with smartphones to enhance blood pressure regulation in patients post-myocardial infarction. This technology also reduced the workload of doctors and automatically generated follow-up reports by integrating patient health records, thereby improving patient compliance (147). Johnson et al. implemented a chronic disease care model focused on depression in India. This model improved the care of chronic disease patients through multidisciplinary team collaboration, patient self-management education, telemedicine, and digital management. The model emphasized holistic care, coordinated care, accessible services, quality and safety, and enhanced relationships between medical providers and patients (148). Subramanian et al. utilized artificial intelligence in the realm of chronic diseases, focusing on health monitoring and management, electronic health records, and the transformation of chronic disease management models. This was achieved by collecting pertinent indicator values via patient-worn monitoring devices and adhering to predefined algorithms. Consequently, professional recommendations were offered to patients, leading to an enhancement in patient satisfaction (149).

The integration of AI and IoT technologies not only amplifies patients' awareness and engagement in disease management but also furnishes medical professionals with robust tools to more efficiently support the self-management and health enhancement of chronic disease patients. As technology continues to evolve, it is projected that these tools will assume an increasingly significant role in the management of chronic diseases.

## 4 Architecture of a chronic disease monitoring management platform based on IoT mobile sensing device data and machine learning algorithms

### 4.1 Platform architecture overview

**Figure 2:**
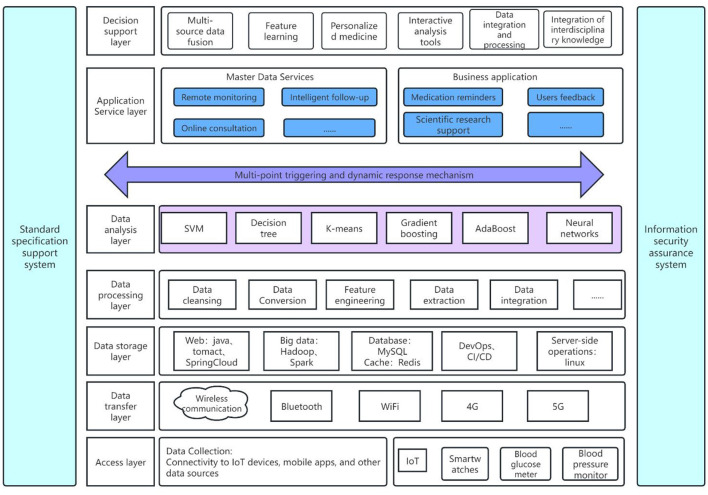
Architecture of chronic disease monitoring management platform based on IoT mobile sensing device data and machine learning algorithms.

The design of a system architecture for a chronic disease monitoring management platform, leveraging IoT mobile sensing device data and machine learning algorithms, can be segmented into multiple layers, each performing distinct functions, as shown in Figure 2.

The access layer is primarily tasked with establishing connections with IoT devices, mobile applications, and other data sources. This encompasses wearable and mobile devices such as smartwatches, health trackers, glucometers, blood pressure monitors, among others. These devices have the capacity to gather patients' physiological data in real-time, including heart rate, blood pressure, blood glucose levels, and activity levels. Salehi et al. employed assistive mobile health applications and IoT-based wearable devices for the continuous health monitoring of Alzheimer's patients. This approach not only alleviates the strain on healthcare systems but also reduces operational costs while enhancing the quality of life for Alzheimer's patients (150).

The data transmission layer, which gathers information from the access layer, employs wireless communication technologies such as Bluetooth, WiFi, and 4G/5G to relay data to servers or cloud platforms. Huang et al. developed a healthcare system framework that captures chronic disease medical data from IoT and wireless body-area networks. This data is then transmitted via a comprehensive wireless sensor network infrastructure and published to a wireless personal area network through a gateway, thereby improving data transmission efficiency (151). Allahham et al. introduced an Intelligent, Secure, and Energy-efficient (I-SEE) framework that utilizes a Deep Deterministic Policy Gradient (DDPG) algorithm, specifically the Static-DDPG, to ensure the efficient transmission of large volumes of private chronic disease data. This approach achieves secure and energy-efficient medical data transmission (152). França et al. employed discrete event modeling, specifically the Coding of Bits for Entities by Discrete Events (CBEDE), to construct an AWGN communication channel model with Differential Quadrature Phase Shift Keying (DQPSK) modulation. They analyzed the correlation between the information consumption of medical data in megabytes (MB). This approach significantly improves the transmission capacity within healthcare systems, enabling more efficient and rapid scheduling of consultations and monitoring of patient data (153).

The data storage layer employs database management systems, including Structured Query Language (SQL) and Not Only SQL (NoSQL) databases, as well as data warehousing technologies to store processed data. This provides a solid foundation for subsequent analysis and decision support. Saranya et al. developed an intelligent healthcare data warehousing and mining system that enhances the efficiency of data storage, indexing, and sharing by assigning corpus-aware medical terms to medical records. This system offers effective medical services to health seekers (154). Shuli et al. proposed a combined storage solution that utilizes RELATION databases and native XML databases with a fine-grained EMR data structure. This approach effectively reduces EMR data storage space and query time complexity, thereby facilitating researchers in later stages for medical information statistics and clinical diagnosis (155).

The data processing layer utilizes various preprocessing techniques, including data cleaning, format conversion, and outlier handling, to integrate and conduct preliminary analysis on the collected raw data. Chiang et al. performed an in-depth data cleaning and phenotype analysis based on electronic medical records, quantifying the performance of different phenotype algorithms in identifying infective endocarditis. This was done with the aim of enhancing diagnostic efficacy and accuracy in mortality assessment (156). Similarly, Phan et al. developed and optimized a protocol for cleaning pediatric height and weight data. They employed robust linear regression for outlier detection, which allowed for the automatic cleaning of anomalies in longitudinal electronic health records. This successfully standardized data quality for clinical and research applications related to obesity, hypertension, type 2 diabetes, and malnutrition (157). Lin et al. used growthcleanr to identify outliers in height and weight trajectories and errors in pediatric and adult electronic health records. These errors included unit errors, incorrect figures, duplicates, and carry-over values. The researchers compared growthcleanr with other common pediatric data cleaning algorithms. The results indicated that growthcleanr is the only method capable of cleaning adult data and identifying pervasive carry-over and duplicate data in EHRs (158).

The data analysis layer employs statistical analysis, predictive models, clustering analysis, and other machine learning algorithms to conduct an in-depth examination of stored data. This process extracts valuable information such as disease prediction, health trend analysis, and anomaly detection. Zolbanin et al. utilized these data analysis methods to develop and test deep neural networks for predicting patient hospitalization times and analyzing patient health trends. This approach effectively optimized resource utilization, such as bed occupancy, to maximize revenue (159). Davagdorj et al. proposed an efficient framework based on Extreme Gradient Boosting (XGBoost), which was combined with a Hybrid Feature Selection (HFS) method for predicting SiNCDs in the general populations of South Korea and the USA. Empirical analysis demonstrated that the proposed model outperformed existing baseline models (160). Utomo et al. proposed a machine learning model, utilizing the CatBoost algorithm, for diagnosing diabetes based on 24-h data from Intensive Care Unit (ICU) patients. The efficacy of this method was validated through experiments with real medical data and comparative analysis against various other machine learning algorithms. The results indicated that the proposed method outperformed all baseline methods in terms of performance (161).

The application service layer offers specialized services including patient education, knowledge dissemination, intelligent follow-ups, medication reminders, and health record management. Zhang et al. introduced an ontology-based framework that amalgamates data on type 2 diabetes patients with medical domain knowledge and patient assessment criteria to facilitate chronic disease patient follow-up evaluations. They subsequently developed a clinical decision support system to operationalize this framework, which autonomously selects and modifies standard assessment protocols to align with individual patient conditions, thereby enhancing the accessibility, efficiency, and quality of current type 2 diabetes follow-up services (162). Wardhani et al. developed a medication reminder system for chronic disease patients utilizing IoT technology. This system interfaces with a medication reminder device constructed using Raspberry Pi 3 Model B and features IoT-based database monitoring. The system enables real-time monitoring and significantly enhances medication adherence levels among chronic disease patients (163).

The decision support layer employs data analysis outcomes to furnish personalized treatment recommendations and health management strategies, thereby offering healthcare professionals with the necessary decision-making tools to devise more effective treatment and management approaches. Wu et al. investigated a mobile healthcare system that utilizes efficient data decision-making and wireless network communication to aid older adult individuals in accessing remote medical services. They introduced a deep learning model, the Combination Sparse Autoencoder (CSAE), into the decision module. This model employs sparse autoencoders to concurrently process patient detection and monitoring data, thereby achieving the association and integration of multi-source data. Disease prediction probabilities are derived through deep neural networks and classifiers, resulting in the optimal disease prediction sequence for chronic disease prediction and early warning (164). Alsuhibany et al. developed a deep learning-based clinical decision support system (EDL-CDSS) that incorporates ensembles of deep belief networks, kernel extreme learning machines, and gated recurrent unit CNN for CKD diagnosis in an IoT environment. The objective of EDL-CDSS technology is to detect and classify different stages of CKD using healthcare data collected from IoT devices and benchmark repositories (165). Ali et al. proposed a novel medical monitoring framework based on cloud environments and big data analysis engines. Their big data analysis engine, which integrates data mining techniques, ontologies, and bidirectional long short-term memory (Bi-LSTM), effectively preprocesses healthcare data and reduces its dimensionality. Bi-LSTM accurately classifies medical data, such as diabetes, to predict medication side effects and anomalies (166).

Safety and privacy protection layers are implemented through data encryption, access control, user authentication, and privacy protection policies to ensure both the security of data and the privacy of patients. Elhoseny et al. proposed a secure model for medical data transmission in an IoT-based healthcare system. This model combines 2-D discrete wavelet transform level 1 or 2-D discrete wavelet transform level 2 steganography techniques with a proposed hybrid encryption scheme to protect diagnostic text data within medical images. The model is capable of concealing confidential patient data within the transmitted cover image, offering a high degree of imperceptibility, capacity, and minimal degradation of the received steganographic image (167).

In the realm of chronic disease monitoring management platforms, the establishment of a standardized support system is paramount. This ensures data consistency, system interoperability, service quality, and security. Pradeepa et al. employ standardized communication protocols, such as the Message Queuing Telemetry Transport (MQTT) protocol, to monitor vital signs like blood pressure. This facilitates efficient data exchange between devices, systems, and services (168). Adhering to healthcare industry regulations and standards, including the Health Insurance Portability and Accountability Act (HIPAA), the Economic and Clinical Health Information Technology Act (HITECH Act), and the 21^st^ Century Cures Act, is essential for platform compliance (169). Furthermore, the adoption of standardized medical terminology and coding systems, such as ICD and SNOMED CT, is crucial for supporting accurate medical records and analysis (170).

This hierarchical architectural design enables the chronic disease monitoring management platform to facilitate comprehensive, continuous health management services for patients with chronic diseases. This is achieved through a full-process management system that encompasses data collection and decision support.

### 4.2 Key IoT components and functions

IoT mobile sensing devices are increasingly utilized in the collection of daily activity data for chronic disease patients. These devices enable medical professionals and patients to gain a better understanding of health conditions by monitoring daily activities and implementing corresponding intervention measures. Wu et al. developed a compact wearable sensor patch designed to measure various physiological signals, including electrocardiograms, photoplethysmograms, and body temperature. All components of this device utilize a combination of rigid and flexible structures, making it easy to attach to the human body for remote health monitoring applications (171). Valero-Ramon et al. employed process mining techniques to uncover dynamic risk models for chronic diseases such as hypertension, obesity, and diabetes. These models were based on the dynamic behaviors of patients provided by health sensors, aiming to improve chronic disease management (172). Awotunde et al. proposed an IoT-based WBN machine learning algorithm framework. This framework collects data from various wearable sensors (such as body temperature, blood glucose sensors, heart rate sensors, and chest) via IoT devices and transmits them to an integrated cloud database. Machine learning is then used to analyze sensor signals for patient data diagnosis. This framework has the potential for widespread use in remote areas to monitor and diagnose the health status of patients. It could reduce and eliminate medical failures, lower medical costs, and enhance patient satisfaction (173). Sangeethalakshmi et al. proposed an IoT-based real-time health monitoring system, comprising a mobile application and GSM, designed for the regular monitoring, display, and storage of patients' vital signs such as body temperature, heart rate, electrocardiogram, blood pressure, and SPO2. This system enables healthcare professionals to monitor hospitalized or home-based patients using the integrated IoT-based healthcare system, thereby ensuring high-quality patient care (174). In response to the increasing number of chronic disease patients in the Netherlands, Medicine Men developed an electronic health solution called Emma Activity Coach. This solution operates on Fitbit smartwatches, enabling chronic disease patients to monitor their activities in real-time with the support of informal caregivers and healthcare professionals, thereby improving their quality of life (175). Kim developed a prediction model using SVM that utilizes easily measurable health-related data from smartwatch users to predict the incidence of cardiovascular diseases. This enhances chronic disease patients' ability to self-monitor their health status and personal activities (176).

Environmental health sensors play a crucial role in assessing the health of chronic disease patients, given that environmental factors such as air quality, temperature, and humidity directly influence their health status. Haghi et al. developed an innovative wrist-worn prototype for environmental monitoring, which includes a flexible IoT gateway. This prototype measures key parameters within the environmental domain. The platform facilitates real-time two-way communication between end-users and medical personnel via the IoT gateway, which serves as an intermediary hub between wearable devices and IoT servers. This system allows for early detection of disease functions, thereby enabling prediction and prevention (177). Saini et al. reviewed the use of microcontrollers in system design and the development of real-time monitoring systems. They used wireless technology to develop an indoor environmental quality monitoring system for real-time monitoring of indoor air pollution, with the aim of reducing mortality and morbidity rates caused by this pollution (178). Asha et al. designed an IoT-based Environmental Toxicology and Pollution Monitoring using Artificial Intelligence Technology (ETAPM-AIT) to enhance human health. The proposed ETAPM-AIT model incorporates an array of IoT-based sensors for detecting eight pollutants: NH3, CO, NO2, CH4, CO2, PM2.5, temperature, and humidity. The model aims to use cloud servers to report real-time air quality conditions and issue alerts when harmful pollutant levels are detected. For the classification of air pollutants and determination of air quality, an Elman Neural Network (ENN) model based on an artificial algae algorithm is employed as a classifier. This model can predict future air quality, thereby improving the health status of chronic disease patients (179). Yang et al. explored the sensing mechanism and construction principles of luminescent metal-organic frameworks with adjustable structures, with a particular emphasis on advanced luminescent sensors for environmental pollutants. These sensors are based on metal-organic frameworks and are capable of detecting a range of harmful substances, including pesticides, antibiotics, explosives, volatile organic compounds (VOCs), toxic gases, small toxic molecules, radioactive ions, and heavy metal ions. The effective detection of these substances can significantly reduce the incidence of diseases such as organ failure, deformities, vascular diseases, and cancer (180). Table 3 provides a comprehensive overview of key IoT sensors and their functional principles, highlighting their applications in monitoring and managing various chronic diseases.

**Table 3:** Functional principles of key IoT sensors in chronic disease management.

**Sensor**	**Functional principle**	**Disease**	**References**
Accelerometers, gyroscopes and magnetometer	Analyze and interpret motion data, including wrist and body movements.	Parkinson's disease	(240)
Wearable inertial motion sensor	The objective of this study is to identify variations in acceleration during human movement, with the aim of monitoring activity levels, gait patterns, and tremors. These variations will subsequently be converted into kinematic and kinetic parameters for further analysis.	Parkinson's disease	(241)
Electrocardiogram sensor	These sensors are designed to detect the minute mechanical vibrations produced during heart contractions and relaxations, subsequently converting them into electrical signals.	Heart disease	(242)
Flexible pressure sensor	Pulse wave signals are scrutinized by detecting diastolic alterations in blood vessels, utilizing flexible pressure or strain sensors.	Cardiovascular disease	(243)
Noninvasive glucose-monitoring device	Interstitial fluid glucose concentrations are continuously monitored using subcutaneous sensors, while blood glucose data and trends are relayed through wireless transmission devices.	Diabetes	(244)
Glucose sensor	The process involves the selective identification and measurement of HbA1c or other related substances in blood, utilizing either electrochemical methods or immunoaffinity.	Diabetes	(245)
Wearable electrochemical glucose sensor	When affixed to human skin, this device consistently measures sweat glucose levels and conducts comprehensive glucose analysis.	Diabetes	(246)
Microcavity assisted graphene pressure sensor	The monitoring of single-vessel local blood pressure is facilitated by gas pressure buffering.	Hypertension	(247)
Wearable ultrasound blood pressure sensor	The transmission and reception of ultrasound, utilizing the reflection and Doppler effect within the blood vessel wall and blood flow rate, facilitates the measurement of dynamic changes in blood vessels.	Hypertension	(248)
Microneedle coupled epidermal sensor	Microneedles possess the capability to penetrate the stratum corneum, facilitating the rapid extraction of interstitial fluid onto the sensors.	CKD	(249)

Several databases were searched for relevant literature, including PubMed, Web of Science, Scopus, and IEEE Xplore. We have a selection of the keywords and search terms that were used to identify relevant studies, including “IoT in healthcare,” “chronic disease management,” “machine learning algorithms,” “mobile sensing devices,” and “AI in medicine.”

### 4.3 Multi-point trigger mechanism and dynamic response

By integrating a sophisticated rule engine with machine learning algorithms, the chronic disease management platform is capable of dynamically monitoring and conducting real-time analysis of patients' health status.

**Figure 3:**
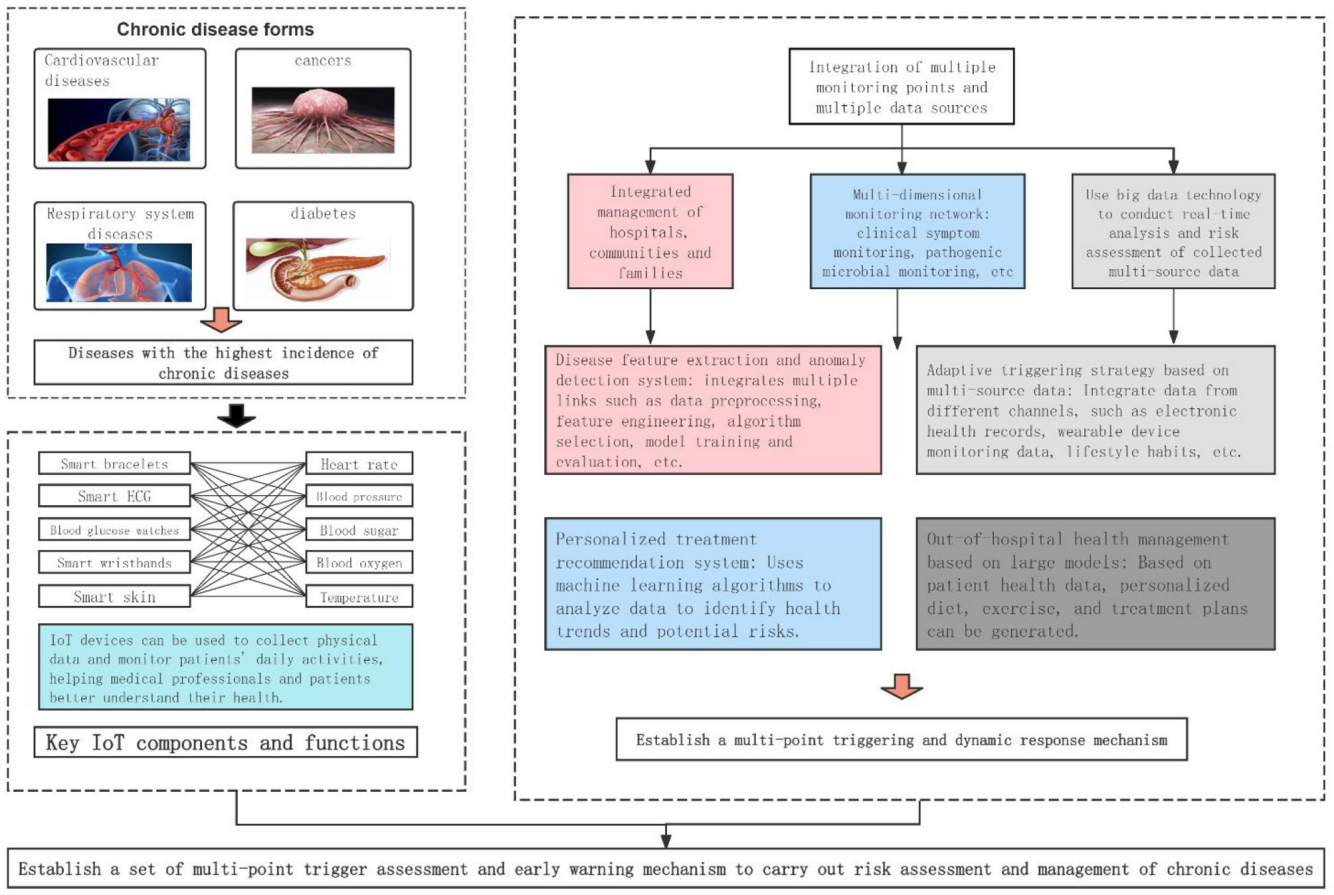
Multi-point triggering mechanism and dynamic response framework.

The design of the multi-point triggering mechanism and dynamic response framework is depicted in Figure 3.

The Disease Feature Extraction and Anomaly Detection System leverages real-time data from IoT devices in conjunction with historical health records. This system employs feature extraction and anomaly detection algorithms to identify any deviations from standard patterns, thereby facilitating a swift response to potential health alterations. The process of feature analysis necessitates the identification of variation patterns in time-series data derived from historical monitoring indicators, such as periodicity, drift, and stationarity, to select the most suitable algorithms. For example, autocorrelation plots can be used to identify periodic changes, while median smoothing and trend component detection can capture drift. Stationarity changes are assessed using unit root tests to determine if the time series is stationary. When selecting an algorithm, it is essential to choose the appropriate anomaly detection algorithm based on the data's distribution characteristics. Commonly utilized algorithms in this field include box plots, absolute median differences, and extreme value theory, among others. These algorithms are capable of managing various data distributions, thereby effectively identifying anomalies. Two crucial aspects of system design include model training and real-time detection. Real-time detection typically employs stream processing technologies such as Flink for online anomaly detection, while offline training involves reading training data from a data warehouse, training the model, and saving it for use in real-time detection. Machine learning techniques also play a significant role in the identification and prediction of chronic diseases. By utilizing algorithms such as CNN for automatic feature extraction and the KNN algorithm for precise matching, the accuracy of chronic disease identification can be significantly improved. Kai Wang et al. proposed a novel method for applying deep learning in physiological signal analysis, enabling physicians to identify potential risks. A CNN model was applied using an unsupervised feature learning method to learn meaningful feature representations from unlabeled physiological signals. By leveraging deep learning principles, non-linear, unsupervised, and multivariate Gaussian distribution model methods can be used to eliminate the limitations of current feature extraction and feature selection in physiological signal anomaly detection. The convolution and pooling of CNN can process high-dimensional data more quickly, using features extracted from the CNN source to the anomaly detection model, and providing anomalous data to physicians for assessing the risk of disease before it occurs (181). Ramesh et al. proposed an end-to-end remote monitoring framework, which involved the development of a SVM for diabetes risk prediction using the Pima Indian Diabetes Database. They addressed missing data by imputing features, standardized the range of dataset values through scaling, selected features to eliminate redundancy with minimal contribution to the prediction outcome, and augmented features to correct class imbalance. This process facilitated automated diabetes risk prediction and management (182). Chen et al. introduced an Adaptive Hybrid Deep Convolutional Neural Network (AHDCNN) for early kidney disease detection. Each convolutional layer encompasses three stages: spatial max pooling, group normalization ReLU gating, and linear convolution. For each CNN image input, the output of each layer is extracted to form hierarchical image features. The AHDCNN can achieve accurate segmentation of the integrated system through smoothing and prior knowledge (183). Akter et al. implemented seven advanced deep learning algorithms (ANN, LSTM, GRU, bidirectional LSTM, bidirectional GRU, MLP, and Simple RNN) for the prediction and classification of CKD. They fitted the model to the dataset to identify the most suitable algorithm for analysis, applying multiple input and output layers, different activation functions, and various parameters to compile and fit the model. Perceptron, Adaboost, and random forest classifier models were also employed, successfully identifying nine significant risk factors for CKD, including erythropoietin (184). Du et al. employed ensemble methods, specifically XGBoost, to construct a high-precision model for predicting Coronary Heart Disease (CHD). Following feature processing, 65 feature variables were selected as input for machine learning algorithms. The model was trained, validated, and tested on separate training and test datasets. Notably, the linear ensemble method XGBoost demonstrated superior accuracy on the test dataset (185). Praveen Ramalingam et al. introduced an AI-based heart disease detection system that utilizes machine learning techniques such as random forest. This algorithm's random forest classification operates by constructing multiple decision trees. It assigns weights to the potential reduction of impurity at nodes and determines feature significance by evaluating node hits to enhance impurity (186). Alotaibi et al. introduced Sehaa, a comprehensive big data analytics system tailored for healthcare in Saudi Arabia. The data collection module employs the Twitter stream API to capture and archive public tweet messages, focusing on diseases such as skin conditions, heart disease, hypertension, cancer, and diabetes, all within predefined parameters. The preprocessing module meticulously cleans and manually labels this data, setting the stage for subsequent learning and classification phases. The classification module is comprised of six distinct models across two categorization stages. Employed classification techniques encompass Naive Bayes and logistic regression, in conjunction with four feature extraction methods: BiGram, TriGram, HashingTF, and CountVectorizer. These methods aim to predict the category, class, or label of input data based on rules derived during the learning or training phase. In the learning phase, the classifier leverages labeled data (training data) to formulate classification rules, thereby learning how to predict future data labels (187).

An adaptive triggering strategy, based on multi-source data, is proposed. This strategy involves real-time monitoring of patients' health statuses by integrating data from various channels such as electronic health records, wearable device monitoring data, lifestyle habits, and biomarkers. The comprehensive health profile established for each patient is then transmitted to a central processing system, thereby creating an intelligent multi-point triggering disease prediction and assessment system. Altenbuchinger et al. introduced a data integration framework that adjusts for confounding variables. This multi-source data integration method revealed significant associations between CKD comorbidities and metabolites, including a novel association between the plasma metabolite trimethylamine-N-oxide and arrhythmias and infarctions in patients with stage 3 CKD (188). Zachariou et al. developed a network-based integrated approach that maximizes the number of data sources. This system-level method can provide holistic insights for disease risk assessment, disease onset and progression prediction, effective treatment, identification of potential drug targets, and computational drug discovery and repurposing related research questions for diseases such as Alzheimer's (189). Wang et al. introduced the concept of Multiple Hybrid Attribute Information Systems (MHAISs), applying the proposed method to assess the risk of hypertension. They constructed a variable precision multi-granularity kernel rough set (VPMGKRS) and proposed a MHAIS-based multi-granularity three-way decision-making method. This method takes into account individual differences among decision-making objects and the diversity of features between data sources. The loss function at different granularities is calculated using conditional probability, thereby obtaining the threshold for three-way decision-making (190). Sidorenkov et al. developed multi-source predictive models for participant selection and nodule management, both pre and post baseline screening. They constructed and optimized polygenic risk scores and air pollution-based environmental risk scores to predict lung cancer. The focus of their work was on integrating multi-source data from various domains. Both static (such as genetic) and dynamic risk markers (such as imaging, environmental, and behavioral markers) were integrated, not only for baseline screening but also for ongoing screening (191). Li et al. employed a Bayesian conditional autoregressive spatial model, using multi-source data summarized in 1,025 communities in China to explore the BE-health relationship. They conducted a systematic community-level modeling using a diverse range of BE factors such as community attributes, urban form and configuration, facilities, landscape, and location. This allowed them to identify a set of community built-environment factors that affect the risk of ischemic heart disease (192). Alramadeen et al. introduced a sparse linear mixed model designed for the remote monitoring and diagnosis of sleep disorders. This model integrates the modified Cholesky decomposition with the group lasso penalty, facilitating joint group selection of both fixed and random effects. Additionally, it synergizes the expectation-maximization algorithm with an optimized specialized maximization technique (193).

A personalized treatment recommendation system leverages machine learning algorithms to analyze data, accurately discern individual health trends and potential risks, and dynamically adjust chronic disease management plans. The model training ensures that each patient's treatment plan is tailored. By analyzing patients' health data, the system offers personalized dietary, exercise, and medication treatment recommendations to assist patients in better managing their conditions (194). Establishing effective feedback channels allows these responses to be used to further optimize algorithms and enhance services (195). A closed-loop feedback mechanism enables the system to update and optimize treatment plans in real-time based on the patient's actual response and health changes (196). Machine learning algorithms, such as random forests or autoencoders, are employed to promptly capture anomalies in data and trigger early warning systems. These patterns may indicate a change in the patient's health status (197). Intelligent recommendation systems provide the most suitable adjustment suggestions based on the patient's specific situation. Furthermore, the strategy employs charts and graphics to visually display health data and trends, enabling patients to quickly comprehend their health status and progress (198). This emphasizes the intuitiveness and ease of use of human-computer interaction, supports multidisciplinary team collaboration, and encourages patients to actively participate in their own health management (199). The system's design incorporates compliance, ethics, privacy protection, and the integration and scalability of technology to ensure the safety and efficacy of adaptive response strategies. Hussein et al. proposed a method for a chronic disease diagnosis recommendation system based on a hybrid approach of multi-classification and unified collaborative filtering. This involves applying multi-classification using decision tree algorithms to construct an accurate prediction model for diagnosing disease risks in monitored cases. When recommending medical advice to patients, higher accuracy can be achieved by using learning classification models based on historical binary ratings and external features (200). Alian et al. proposed a self-care recommendation system for diabetes that recommends a healthy lifestyle to users to combat the disease. By integrating the ontological characteristics of AI users with general clinical diabetes recommendations and guidelines, the system can provide personalized recommendations for AI patients, taking into account social, economic, cultural, and geographical conditions (e.g., food intake and physical exercise) (201).

Outpatient Health Management Utilizing Large Models: Large models can function as virtual health assistants, offering information and guidance pertaining to chronic diseases. This aids patients in gaining a deeper understanding of their conditions. Leveraging the health data supplied by patients, these models can formulate personalized dietary, exercise, and treatment plans. Patients can engage with these models, documenting changes in symptoms and lifestyle habits. The model then assists in monitoring the progression of chronic diseases. Integrated chatbots or virtual assistants offer emotional support and encouragement, helping patients manage the psychological stress induced by chronic diseases. This enhances patients' sense of involvement and control (202). Al-Anezi employed the Chat Generative Pre-trained Transformer (ChatGPT) as a virtual health coach for chronic disease management. This approach significantly enhances the sustainability of healthcare operations, particularly in managing significant diseases (203). Cankurtaran et al. evaluated the efficacy of ChatGPT in the context of inflammatory bowel disease, suggesting that it can serve as a dependable and beneficial resource for both patients and healthcare professionals (204). Mondal et al. employed an AI-based large language model to generate personalized queries for users. The findings revealed that this model could function as a virtual telemedicine agent, providing highly accurate information in most instances. It effectively addressed queries related to lifestyle-related diseases or disorders such as obesity, diabetes, cardiovascular health, and mental health. Therefore, when patients have limited time to consult a doctor or wait for an appointment to seek advice on their condition, they can obtain preliminary guidance (205). Uz and Umay utilized a seven-point Likert scale to assess the reliability and usefulness of ChatGPT in obtaining information about common rheumatic diseases, including osteoarthritis, rheumatoid arthritis, ankylosing spondylitis, systemic lupus erythematosus, psoriatic arthritis, fibromyalgia syndrome, and gout. The results indicated that the model is reliable and useful for patients seeking information on rheumatic diseases (206). Li et al. incorporated advanced AI technology in voice recognition for creating electronic health records and text mining. They constructed a prototype of a “doctor assistant” to record conversations between doctors and patients, which were then converted into text files. Through text mining technology, key symptoms described by patients were abstracted and transformed into structured data. This significantly reduced the workload of doctors and improved the accuracy and quality of electronic health records (207). The Family Doctor Follow-up Center at Beijing Health Hospital employed voice templates, developed with iFlytek's intelligent voice technology, to execute robot call-out services. These services encompassed contracted inpatient physician physical examination appointments and notifications, hypertension patient follow-up management, and hospital patient satisfaction surveys, among others. Empirical evidence suggests that the intelligent call-out platform not only reduces the cost of manual calls but also enhances call quality. Furthermore, it augments the health management efficiency of the family doctor team and the efficacy of hypertension management. This leads to improved patient experience and hospital management standards, ultimately promoting the wellbeing of residents (208).

## 5 Chronic disease medical image reconstruction

### 5.1 Medical image segmentation and denoising

Medical image segmentation and denoizing play pivotal roles in medical image processing, significantly enhancing diagnostic accuracy and efficiency. These methodologies are designed to distill meaningful data from intricate medical images, such as tissue configurations and lesion sites, while simultaneously eliminating image noise to elevate image quality. In recent years, a plethora of researchers have dedicated their efforts to the development of innovative algorithms and methods to refine these techniques.

In 2013, Cai et al. introduced a tight frame-based approach for the automatic identification of tubular structures in medical imaging, primarily targeting vessel segmentation in magnetic resonance angiography images (209). This technique operates by iteratively refining regions that encompass potential vessel boundaries. Concurrently, Zhou et al. explored sparse regularization-based methods for medical image reconstruction and devised an adaptive beam tight frame method for this purpose (210). Their approach begins with the construction of task-specific adaptive beam tight frames, which are then employed to address the l(1)-regularized minimization problem, facilitating the reconstruction of the desired image.

In 2015, Liu et al. introduced a data-driven tight frame magnetic resonance image reconstruction method (DDTF-MRI), which employs an adaptive tight frame to sparsely represent the MR image under reconstruction (211). Concurrently, they devised a two-level Bregman iterative algorithm to address the proposed model. Subsequently, in 2017, another study presented a novel MR image reconstruction model termed the adaptive tight frame and total variation MR image reconstruction model (TFTV-MRI) (212). This model integrates adaptive tight frame learning with total variation for enhanced image reconstruction.

The 2018 study concentrated on the advancements in PET and MRI scanner technology, introducing a robust frame-based joint reconstruction model for PET-MRI that leverages the joint sparsity of tight frame coefficients (213). To address the challenges posed by the non-convex and non-smooth nature of this model, a proximal alternating minimization algorithm was suggested. In the subsequent year, Chan et al. put forth an efficient variational approach for segmenting images characterized by intensity in homogeneities (214). This method is bifurcated into two phases: initially, the image is segregated into reflectance and illumination components through a convex energy minimization model; subsequently, the original image is reconstructed by thresholding the reflectance component.

In 2024, Wu et al. meticulously employed a multilevel wavelet convolutional neural network (MWCNN) to address the challenge of medical image reconstruction, aiming to preserve intricate details while striking an optimal balance between computational efficiency and numerical performance (215). Concurrently, Wang et al. introduced an innovative MRI reconstruction algorithm predicated on image decomposition, specifically tailored to mitigate the slow imaging speed inherent in MRI systems, thereby facilitating precise image reconstruction from undersampled k-space data (216).

### 5.2 Magnetic resonance imaging reconstruction

MRI is a non-invasive medical imaging technology extensively utilized in clinical diagnosis and research. However, the time-consuming and costly data acquisition process associated with MRI has consistently made the enhancement of reconstruction speed and quality a focal point of research. In recent years, the application of Compressed Sensing (CS) theory and tight frame methods in MRI reconstruction has become increasingly prevalent (217). These methods leverage the sparsity of images to reconstruct high-quality MR images from significantly undersampled K-space data (218). Several studies have suggested the use of adaptive tight frame-based MRI reconstruction methods. These incorporate adaptive tight frames into MRI reconstruction by addressing the L0 regularization minimization problem, thereby significantly enhancing the reconstruction speed while maintaining a performance comparable to overcomplete dictionary-based methods (219). Additionally, some research has proposed data-driven tight frame and total generalized variation-based compressed sensing MRI methods (220). These adaptively learn a set of filters from undersampled data, providing a superior image sparse approximation while avoiding staircase effects (221, 222). Furthermore, certain studies have proposed continuous domain regularization-based compressed sensing MRI reconstruction methods, which are based on finite innovation rates (223). These methods achieve a sparse representation of images by assuming that the discontinuities or edges of images are located in the null sets of bandlimited periodic functions.

### 5.3 Computed tomography reconstruction

CT reconstruction serves as a pivotal technique in both medical imaging and industrial non-destructive testing. This method operates by acquiring projection data from various angles of an object, subsequently employing mathematical algorithms to reconstruct the object's cross-sectional images. In recent years, the amplification of computational power coupled with advancements in algorithmic approaches has significantly propelled the development of CT reconstruction technology. This progress is particularly evident in the reduction of radiation dose, enhancement of image quality, and resolution of limited angle challenges.

In 2013, Zhao et al. explored the iterative reconstruction technique based on tight frame (IRIR) for spectral breast CT, aiming to enhance image quality with limited projection data (224). In 2016, a study introduced a spatial-Radon domain CT image reconstruction model using data-driven tight frame (SRD-DDTF) (225). This model integrates the concepts from Dong et al.'s joint image and radon domain painting model with the data-driven tight frame for image denoising. Distinctively, the SRD-DDTF model simultaneously reconstructs both the CT image and its associated high-quality projection image. In 2018, an algorithm was proposed that leverages wavelet tight frame and l(0) quasi-norm to address the exterior CT problem, aiming to minimize artifacts and produce high-quality images (226). That same year, another study introduced an adaptive reconstruction method for the limited angle CT problem, employing both total variation (TV) and data-driven tight frame-based spatial and Radon domain regularization models concurrently (227). Additionally, there was a proposal for an image reconstruction approach that merges TV and wavelet tight frame techniques for the limited angle CT challenge (228). In 2019, Kong et al. introduced an innovative algorithm utilizing tight frame wavelets and total variation for spectral CT (229). By 2020, Goudarzi et al. unveiled a groundbreaking method employing generative adversarial networks (GAN) in ultrasound imaging, targeting superior axial resolution without compromising imaging depth (230). Finally, in 2021, a study put forth a novel CT image reconstruction model predicated on non-local low rank regularity combined with data-driven tight frame (NLR-DDTF) (231). Upon comparing these papers, it is evident that they share a common goal of enhancing the image quality of CT reconstruction, minimizing artifacts, and addressing challenges such as limited angle. They uniformly employ tight frames, total variation, or other regularization techniques to bolster the reconstruction outcome. However, each paper introduces distinct methods and algorithms tailored to specific problems or application contexts. For instance, some papers concentrate on spectral CT, others on limited-angle CT, and some on ultrasound imaging. Furthermore, the techniques and methodologies they utilize vary, including wavelet tight frames, data-driven tight frames, and generative adversarial networks. In summary, while these papers have all significantly advanced the field of CT reconstruction technology, their methodologies and focal points differ.

### 5.4 The utilization of tight frames in medical imaging

Medical image processing constitutes a crucial aspect of medical imaging, wherein the Tight Frame method significantly contributes to the restoration and reconstruction of medical images. The Tight Frame is a mathematical instrument utilized for representing and processing signals or images, distinguished by its superior reconstruction stability. This effectively facilitates the recovery of detailed and structural information embedded in images.

In 2016, Choi et al. examined the two-dimensional reconstruction problem associated with X-ray CT (232). They focused on projections truncated along the spatial direction in the Radon domain and introduced a novel model that demonstrated superior numerical simulation results compared to existing sparsity-based models. Concurrently, another study also addressed the CT's two-dimensional reconstruction issue (233). However, this research attempted to reconstruct the entire CT image from limited data using tight frame regularization and sinogram extrapolation in tandem. This model, too, exhibited more promising numerical simulation results than the prevailing sparsity-based models.

In 2017, a study introduced a novel method for myocardial perfusion-positron emission tomography (MPPET) image recovery within a tight framework (234). This approach leverages the theory of low-rank sparse decomposition to segregate MPPET images and capitalizes on the sparsity of the target image's transformation coefficients within this tight framework. By imposing sparse constraints on the MPPET images, the method effectively recovers them. Experimental evidence suggests that this technique outperforms those that apply constraints in isolation. Concurrently, Luo et al. presented an image restoration algorithm that integrates tight frame wavelets with total generalized variation (228). This algorithm addresses the limitations of conventional total variation regularization techniques, which often result in step-like pseudo-edges and a loss of texture detail in restored images. These studies collectively investigate the utilization of tight frames in medical image processing, with a particular emphasis on image recovery and reconstruction. While they all leverage the theory and methodologies of tight frames to enhance image recovery quality, their specific applications and methodologies vary. For instance, both Choi et al. and another study address the two-dimensional reconstruction challenge in CT scans, but their approaches and focal points differ. Zhang et al. and Liang et al., on the other hand, introduce distinct image restoration algorithms. Furthermore, Tian et al.'s work is primarily centered on devising emergency medical rescue strategies. Collectively, these papers underscore the extensive applicability and significance of tight frames in medical image processing.

## 6 Discussion and future prospects

The technical strengths of the reviewed articles (Specific examples are shown in Table 1):

(1) Advanced model integration: the reviewed articles showcase the integration of cutting-edge models such as deep learning and reinforcement learning within IoT-enabled healthcare systems. These models have demonstrated significant improvements in disease prediction, risk assessment, and personalized treatment plans.
(2) Data-driven approach: a key strength lies in the data-driven approach to chronic disease management. The use of IoT devices for data collection, coupled with machine learning algorithms, allows for a more precise and personalized management of chronic conditions.
(3) Innovative use of technology: the manuscripts reviewed highlight the innovative use of IoT devices and advanced machine learning models, pushing the boundaries of chronic disease monitoring and management and offering new insights into patient care.
(4) Methodological rigor: the reviewed articles employ robust methodologies, such as large-scale multi-center cohort studies and rigorous machine learning algorithms, contributing to the reliability and validity of their findings.

The technical weaknesses of the reviewed articles:

(1) Data collection biases: a common weakness in the reviewed articles is the potential bias in data collected by IoT devices. Inconsistent readings due to variations in sensor sensitivity or user adherence issues can lead to biased health metrics.
(2) Algorithmic limitations: some machine learning algorithms may lack representativeness for specific populations, leading to potential misdiagnoses. Overfitting to training data can result in poor performance when applied to diverse patient populations.
(3) User interaction challenges: the digital divide affects the accessibility and usability of IoT devices among different demographic groups, potentially leading to a biased user base that may not fully represent the broader population.
(4) Data security and privacy concerns: with the increasing use of IoT devices, concerns regarding data security and patient privacy have become more prominent. The reviewed articles may not fully address the need for robust encryption methods and anonymization techniques to protect sensitive health data.
(5) Lack of standardization: the heterogeneity of IoT devices and the lack of standardization in data formats pose significant challenges for data integration and analysis. The reviewed articles do not uniformly propose solutions for ensuring seamless data exchange and interoperability.
(6) Computational resource demands: the growing volume of health data presents a challenge in terms of processing and analyzing this data in real-time, which requires significant computational resources. The reviewed articles may not fully explore the potential role of edge computing and cloud infrastructure in addressing this challenge.

We discuss the potential inaccuracies in data collected by certain IoT devices, which may lead to biased health metrics. For instance, wearable devices can sometimes provide inconsistent readings due to variations in sensor sensitivity or user adherence issues. We highlight the limitations of certain machine learning algorithms that may lack representativeness for specific populations. For example, some algorithms may be overfitted to the training data and perform poorly when applied to a diverse patient population, leading to potential misdiagnoses. We address the challenges in user interaction, such as the digital divide that affects the accessibility and usability of IoT devices among different demographic groups. This can result in a biased user base that may not fully represent the broader population.

With the increasing use of IoT devices, concerns regarding data security and patient privacy have become more prominent. We discuss the need for robust encryption methods and anonymization techniques to protect sensitive health data. The heterogeneity of IoT devices and the lack of standardization in data formats pose significant challenges for data integration and analysis. We highlight the importance of developing uniform standards to ensure seamless data exchange and interoperability. We address the potential for algorithmic bias in machine learning models, which can lead to unfair predictions in disease risk assessment. We emphasize the need for transparent and explainable AI to mitigate these biases. As the volume of health data grows, the challenge of processing and analyzing this data in real-time requires significant computational resources. We discuss the potential role of edge computing and cloud infrastructure in addressing this challenge. The use of AI in healthcare raises ethical questions about decision-making, accountability, and the potential for AI to replace human judgment. We explore the ethical frameworks that can guide the responsible use of AI in chronic disease management. Despite technological advancements, the integration of IoT and AI into clinical practice remains a challenge due to factors such as cost, clinician resistance, and the need for evidence-based outcomes. We discuss strategies to facilitate the adoption of these technologies in healthcare settings. Ensuring that all patients, regardless of their digital literacy or socioeconomic status, can benefit from IoT and AI technologies is a critical challenge. We highlight the need for inclusive design and accessible interfaces to bridge the digital divide.

The potential applications of emerging technologies in chronic disease management are vast, particularly when integrated with quantum computing, biosensors, IoT, and machine learning algorithms. Quantum computing, characterized by its ultra-fast processing speed and capabilities, is revolutionizing the field of medical AI. This technology can efficiently process and analyze large datasets, offering significant value in areas such as drug development, medical big data, and healthcare. For example, in drug development, quantum computing can predict the outcomes of chemical combinations, simulate various precursors formed by different functional groups, rapidly screen promising combinations, thereby reducing research and development time and costs.

Aggarwal et al. introduced a precision-based fine-grained patient diagnostic method, an algorithm that describes the process of patient diagnostic modeling using quantum computing. This involves initializing quantum bits, pairing values, assigning probability values, cross-validation, and forming quantum circuits. The method requires patients to report symptoms, healthcare experts to contact regarding various factors, precise examinations, and a detailed understanding of health status (both past and present medical history). Subsequently, precise interventions are made through the comprehension of biomolecular simulations (264). Enad et al. employed quantum neural networks and quantum SVM to predict heart disease. Their approach aims to enhance the accuracy and speed of identifying various cardiac issues or risk factors by integrating quantum computing concepts into machine learning algorithms. The framework is designed to manage extensive computations and large datasets, ultimately leveraging the scalability and processing power of cloud infrastructure to improve high-definition predictive models. The ultimate goal is to achieve precise diagnostics, provide highly reliable risk assessments, and enable real-time or near-real-time instance classification to improve healthcare diagnostics and mitigation measures in cardiac health monitoring (265). Balamurugan et al. used optimized machine learning models and quantum computing techniques such as Lasso regression for feature selection, Bayesian neural networks for classification, and optical biosensors for cancer cell detection. The accuracy of this research method exceeded that of previously published studies (266). Karthick and Pankajavall developed the IoT-Spiro system, introducing an advanced machine learning prediction framework. This system is capable of detecting various volatile organic compounds present in exhaled breath and analyzing parameters in real time. The proposed framework integrates a hybrid genetic explosion-implosion algorithm for optimal feature selection from real-time datasets, along with a fuzzy-based quantum neural network classifier for the diagnosis of chronic obstructive pulmonary disease (267). Munshi et al. investigated the efficacy of two quantum machine learning algorithms, specifically quantum support vector classifiers and variational quantum classifiers, in predicting chronic heart disease within the context of healthcare 4.0. Their findings indicated that the quantum support vector classifier outperformed the variational quantum classifier, achieving an accuracy rate of 82% (268).

The integration of quantum computing, biosensors, IoT, and machine learning algorithms is poised to significantly enhance chronic disease management. This amalgamation promises to deliver more efficient, precise, and tailored healthcare services. As these technologies evolve and find practical applications, patients with chronic conditions are anticipated to experience improved health management and therapeutic results.

The amalgamation of IoT, AI, and other technologies such as bioinformatics and nanotechnology presents significant potential in the realm of chronic disease management. This is particularly evident in the areas of data collection and analysis.

In the realm of chronic disease management, bioinformatics technologies are employed to scrutinize genetic data, pinpoint disease susceptibility genes, and identify biomarkers. When integrated with biosensor technology, these tools facilitate real-time monitoring of patients' biomolecules, thereby laying the groundwork for personalized treatment approaches. Cao et al. utilized bioinformatics analysis and machine learning to discern biomarkers for diagnosing CKD and non-alcoholic fatty liver disease. Their findings revealed that four genes associated with non-alcoholic fatty liver disease (DUSP1, NR4A1, FOSB, ZFP36) could potentially serve as diagnostic markers for CKD patients complicated by non-alcoholic fatty liver disease. This discovery offers both diagnostic markers and therapeutic targets for CKD patients suffering from non-alcoholic fatty liver disease (269). Zhu et al. conducted a comprehensive bioinformatics analysis and machine learning to screen for immune-related secretory proteins associated with CKD and calcific aortic valve disease. They employed machine learning algorithms, such as Lasso regression and random forest, to identify candidate biomarkers and developed a diagnostic nomogram to predict CKD-related calcific aortic valve disease (270). Zhang et al. delved into the genetic mechanisms of diabetic nephropathy complicated by inflammatory bowel disease using data mining and bioinformatics analysis. Their findings suggest that oxidative stress, chronic inflammatory responses, and immune dysfunction may be the underlying mechanisms for the co-occurrence of diabetic nephropathy and inflammatory bowel disease (271). Lu et al. identified diagnostic biomarkers for idiopathic pulmonary arterial hypertension with metabolic syndrome through bioinformatics and machine learning. They utilized random forest algorithms and the least absolute shrinkage and selection operator algorithm to screen for candidate diagnostic genes at the intersection of differentially expressed genes and weighted gene co-expression network module genes (272).

The incorporation of nanotechnology into drug delivery systems can augment the bioavailability and therapeutic efficacy of drugs, while simultaneously reducing their side effects. Furthermore, nanosensors have the capacity to detect minute changes in biomarkers, thereby facilitating early diagnosis and disease monitoring. Wang et al. employed functionally reconstructed materials and techniques with specific targeting capabilities at the nanoscale level. They posited that drugs enhanced by nanotechnology hold the potential to revolutionize the treatment paradigm for neurodegenerative diseases, while minimizing systemic side effects (273). Chintapula et al. explored nanotech-based immunomodulatory approaches that could potentially be utilized as therapeutic interventions for prominent age-related diseases, including cardiovascular diseases, autoimmune diseases, neurodegenerative diseases, and infectious diseases (274). Shoaib et al. employed biofluid-based glucose sensors, utilizing minimally invasive, invasive, and non-invasive techniques to assess the impact of nanotechnology on biosensors. The benefits of low invasiveness and continuous glucose monitoring in new medical devices can enhance patient comfort and understanding of their blood glucose conditions, thereby improving the healthcare system and overall clinical outcomes (275). Through the integration of these technologies, chronic disease management is progressing toward a more intelligent, personalized, and precise direction, thereby providing patients with superior quality medical services.

The development of smart chronic disease management involves a complex, multi-tiered cooperation model among governments, private enterprises, and medical institutions. This model primarily emphasizes the integration of resources from all stakeholders to collaboratively advance the creation of chronic disease monitoring platforms that utilize IoT and machine learning algorithms.

Governments typically bear the responsibility for establishing pertinent policies and standards, offering financial backing, and providing policy guidance to guarantee the quality and efficiency of medical services. A prime example of this is the “Integration of Medical Services and Prevention” chronic disease service management capacity enhancement project, spearheaded by China's National Health Commission. This project, as part of the “Fifth Round of the Three-Year Action Plan for Public Health,” seeks to establish an integrated and precise model for chronic disease health management, underpinned by information technology. Private enterprises are instrumental in technological innovation, product research and development, and market promotion. They contribute to chronic disease management by developing smart medical devices, health management platforms, and data analysis tools. For instance, Huawei's Smart Health City solution employs cloud computing, big data, AI, and other technologies to create a unified architecture that enables second-level access to images and pathology (276). Medical institutions, as primary service providers, employ IoT devices to gather patient data. They then utilize machine learning algorithms for analysis and formulate personalized treatment plans. These institutions also play a pivotal role in the design and optimization of chronic disease management platforms to ensure they align with clinical requirements. For instance, the Chronic Disease Health Management Support Center, established by the Shanghai Municipal Center for Disease Control and Prevention, employs comprehensive prevention and treatment technologies to precisely collect and monitor comprehensive risk factors for chronic diseases (277).

The Public-Private Partnership (PPP) model holds significant promise for chronic disease management. This model facilitates joint investment, risk sharing, and benefit distribution between governments and the private sector. For instance, the General Office of the State Council has implemented policy measures to expedite the growth of private medical services. These measures encourage societal forces to offer multi-tiered and diverse medical services, while also supporting private medical institutions in collaboration with public hospitals to establish new non-profit medical institutions (278).

In the realm of chronic disease management, technological innovation and enhanced service quality are fundamental to effective collaboration. For example, the Shanghai Medical Care Cloud Information Platform amalgamates medical theories with expert consensus guidelines. By leveraging the Internet, IoT, and AI technologies, it facilitates multi-role collaboration among medical professionals. This platform offers patients comprehensive chronic disease management services, thereby elevating the treatment outcomes for diabetic patients in Shanghai (279). These cooperative models can effectively facilitate the development of chronic disease monitoring management platforms, leveraging IoT and machine learning algorithms. This advancement enables early detection, prevention, treatment, and rehabilitation of chronic diseases, thereby enhancing the quality and efficiency of medical services.

Medical image reconstruction, a pivotal research area in medical imaging, aims to restore comprehensive medical images from incomplete or compromised data. Despite significant advancements in computer technology and algorithmic development in recent years, this field continues to present numerous challenges and opportunities for further exploration. Recent studies have demonstrated the extensive application of Tight Frame technology in medical image reconstruction. This method amalgamates compressed sensing, total variation, and other regularization techniques to effectively address the reconstruction challenges associated with medical images such as MRI and CT. Notably, data-driven Tight Frame methods offer innovative solutions for enhancing image quality and expediting the reconstruction process. Furthermore, the integration of deep learning technology with Tight Frame technology has been explored to further optimize the reconstruction outcomes. Despite significant advancements, medical image reconstruction continues to grapple with several challenges. Firstly, the optimal management of noise and artifacts, along with the acquisition of high-quality images under conditions of low dose or limited angle, remains a focal point of research. Secondly, there is a pressing need for the development of more tailored and efficient reconstruction algorithms specific to various medical imaging modalities and application contexts. Furthermore, in light of the expanding realm of medical big data, leveraging this vast pool of information for model training and optimization has emerged as a pivotal area of inquiry. Looking ahead, we foresee several key trends in medical image reconstruction research. Firstly, the role of deep learning and artificial intelligence technologies is expected to expand, particularly in the automated design and optimization of models. Secondly, there will be increased focus on cross-modality and multi-modality medical image reconstruction to facilitate information fusion and harness the complementary strengths of various imaging techniques. Additionally, real-time and dynamic medical image reconstruction will gain prominence to address the demands of clinical applications. Lastly, as personalized medicine advances, the challenge of tailoring medical image reconstruction to individual differences will emerge as a significant research area. In conclusion, the field of medical image reconstruction is undergoing continuous deepening and expansion. This ongoing research will undoubtedly provide more efficient and accurate imaging support for clinical medicine in the future.

The integration of IoT devices and machine learning algorithms substantially diminishes healthcare costs by curbing unnecessary hospital visits and readmission rates. For instance, the implementation of remote monitoring and prompt interventions can decrease the frequency of emergency room visits and hospitalizations for patients, thereby leading to considerable cost reductions. Through precise forecasting of patient requirements and disease trajectories, healthcare resources can be judiciously allocated, thereby minimizing inefficiencies. For example, machine learning algorithms can identify patients at an elevated risk of readmission, facilitating timely allocation of healthcare resources for preventive interventions. Mitigation of long-term care expenditures: the utilization of IoT devices for the self-management of chronic patients can decelerate disease progression, decrease the necessity for long-term care, and consequently reduce associated costs. Enhancing workflow efficiency: ML algorithms have the potential to automate numerous mundane data processing tasks, thereby enabling medical professionals to allocate more time toward direct patient care and subsequently enhance overall workflow efficiency.

IoT devices offer tailored reminders and suggestions, facilitating patients in more effectively adhering to their treatment regimens. This, in turn, optimizes therapeutic outcomes and bolsters patient compliance. Real-time monitoring and early intervention can more effectively manage the conditions of patients with chronic diseases, thereby reducing acute episodes. This significantly improves the quality of life for these patients. The integration of IoT and ML technologies facilitates access to high-caliber medical services for patients in remote locations, thereby mitigating the disparity between urban and rural healthcare provision and fostering health equity. The integration of IoT devices and applications facilitates patients' comprehension of their health status, thereby actively engaging them in disease management. This, in turn, significantly bolsters the patient's capacity for self-management. Large-scale management of chronic diseases can lead to a reduction in both the incidence and mortality rates associated with these conditions, thereby elevating the overall public health standard within society. The integration of IoT and ML technologies has catalyzed technological advancements within the medical domain, thereby paving the way for the evolution of novel medical devices and therapeutic approaches.

## 7 Conclusion

Our findings not only highlight the technical feasibility of IoT and machine learning algorithms in chronic disease management but also underscore their profound implications for healthcare delivery. The integration of these technologies is anticipated to lead to more efficient resource allocation, reduced hospitalization rates, and enhanced patient self-management capabilities. This approach has the potential to revolutionize patient care by providing personalized treatment plans, improving disease monitoring, and facilitating timely interventions, ultimately leading to better health outcomes and a reduced burden on healthcare systems. The significant economic and social advantages, coupled with the improved quality of life for patients, solidify the position of IoT and machine learning as a transformative model for the future of chronic disease management.

This review underscores the pivotal role of integrating IoT mobile sensing devices with machine learning algorithms and frame theory in chronic disease management and medical image reconstruction. This integration optimizes data collection, enhances disease diagnosis, tailors personalized treatment, and improves disease management and prevention. At a technological level, IoT enables continuous lifestyle and health monitoring, while machine learning excels at disease prediction, risk assessment, and personalization of treatment plans. The synergistic relationship between smart healthcare systems and mobile health applications offers patients personalized nutrition and health management services. It also enhances recovery efficiency through telerehabilitation and enriches patient experiences by promoting engagement, education, and satisfaction. This solidifies the position of IoT and machine learning as a transformative model for chronic disease management, offering tangible improvements in patient care and healthcare system efficiency.
